# Circumventing *Y*. *pestis* Virulence by Early Recruitment of Neutrophils to the Lungs during Pneumonic Plague

**DOI:** 10.1371/journal.ppat.1004893

**Published:** 2015-05-14

**Authors:** Yaron Vagima, Ayelet Zauberman, Yinon Levy, David Gur, Avital Tidhar, Moshe Aftalion, Avigdor Shafferman, Emanuelle Mamroud

**Affiliations:** Department of Biochemistry and Molecular Genetics, Israel Institute for Biological Research, Ness Ziona, Israel; Tufts University, UNITED STATES

## Abstract

Pneumonic plague is a fatal disease caused by *Yersinia pestis* that is associated with a delayed immune response in the lungs. Because neutrophils are the first immune cells recruited to sites of infection, we investigated the mechanisms responsible for their delayed homing to the lung. During the first 24 hr after pulmonary infection with a fully virulent *Y*. *pestis* strain, no significant changes were observed in the lungs in the levels of neutrophils infiltrate, expression of adhesion molecules, or the expression of the major neutrophil chemoattractants keratinocyte cell-derived chemokine (KC), macrophage inflammatory protein 2 (MIP-2) and granulocyte colony stimulating factor (G-CSF). In contrast, early induction of chemokines, rapid neutrophil infiltration and a reduced bacterial burden were observed in the lungs of mice infected with an avirulent *Y*. *pestis* strain. *In vitro* infection of lung-derived cell-lines with a YopJ mutant revealed the involvement of YopJ in the inhibition of chemoattractants expression. However, the recruitment of neutrophils to the lungs of mice infected with the mutant was still delayed and associated with rapid bacterial propagation and mortality. Interestingly, whereas KC, MIP-2 and G-CSF mRNA levels in the lungs were up-regulated early after infection with the mutant, their protein levels remained constant, suggesting that *Y*. *pestis* may employ additional mechanisms to suppress early chemoattractants induction in the lung. It therefore seems that prevention of the early influx of neutrophils to the lungs is of major importance for *Y*. *pestis* virulence. Indeed, pulmonary instillation of KC and MIP-2 to G-CSF-treated mice infected with *Y*. *pestis* led to rapid homing of neutrophils to the lung followed by a reduction in bacterial counts at 24 hr post-infection and improved survival rates. These observations shed new light on the virulence mechanisms of *Y*. *pestis* during pneumonic plague, and have implications for the development of novel therapies against this pathogen.

## Author Summary

The pathogen *Yersinia pestis* is the causative agent of pneumonic plague, as well as a potential bioweapon. The nature of this disease involves an initial non-inflammatory phase where the influx of neutrophils to the lungs is suppressed, allowing bacterial propagation in this organ. Using the mouse model of pneumonic plague, we demonstrate that the early expression of neutrophil chemoattractants and adhesion molecules in the lungs is delayed concomitant with a delayed recruitment of neutrophils to the lung. We also show that the *Y*. *pestis* virulence factor YopJ is involved in the early suppression of chemoattractants mRNA expression in the lung early after infection, but it seems that additional *Y*. *pestis* factors interfere with the protein synthesis of these chemoattractants. Indeed, administration of recombinant KC and MIP-2 to the infected lung of G-CSF treated mice restored the early neutrophil influx to the lungs, leading to a significant reduction in bacterial burden. The treatment has also proved efficacious in reducing mortality. This study highlights the complex virulence mechanisms employed by *Y*. *pestis* to diminish the early homing of neutrophils to the lungs thereby allowing bacterial propagation and disease progression.

## Introduction

The recruitment of neutrophils is a fundamental component of the initial phase of the innate immune response to bacterial lung infections, as demonstrated by the selective depletion of neutrophils and the consequences on pathogen clearance from the lungs [1]. In response to infection, neutrophils are mobilized from the bone marrow (BM), resulting in a rise in circulating neutrophils in the blood within a few hours after infection [2, 3]. The robust expression of G-CSF modulates the production of neutrophils to meet the increased need of the host during infection [4]. Circulating neutrophils migrate to the infection site along a chemotactic gradient of potent chemoattractants, such as KC (CXCL1 or IL-8 in humans) and MIP-2 (CXCL2), produced at the infection site [5, 6]. To allow circulating neutrophils to cross the vascular wall and arrive at the site of infection, multiple adhesion molecules are induced on endothelial cells adjacent to the inflamed tissue. E- and P-selectins are known to be involved in the initial attachment of neutrophils to the endothelium as well as their rolling behavior. Intracellular adhesion molecule 1 (ICAM-1) and vascular cell adhesion molecule 1 (VCAM-1) mediate the subsequent step of tight adhesion to the endothelium, allowing neutrophils to transmigrate to the site of inflammation [7]. After migration, neutrophils phagocytose and digest the invading pathogen and produce pro-inflammatory cytokines [8], thereby serving a beneficial role for the host. However, their excessive and uncontrolled activity may also cause severe damage to the host [9, 10]. The important role of neutrophils in protecting the host against infection with respiratory pathogens has been investigated primarily with regard to pathogens such as *Pseudomonas aeruginosa* [11], *Legionella pneumophila* [12], *Klebsiella pneumonia* [13] and *Yersinia pestis* [14, 15].

*Y*. *pestis* gained notoriety as the causative agent of plague [16]. Inhalation of *Y*. *pestis* droplets or aerosols leads to the development of primary pneumonic plague, which is a rapidly progressing fatal disease with the capability of spreading from person to person [17, 18]. These characteristics also led to the recognition of *Y*. *pestis* as a potential biological threat agent [19].

Recent *in vivo* studies in animal models of pneumonic plague have revealed the biphasic nature of the progression of this disease [20–22]. The observed initial delay in the recruitment of immune cells and neutrophils in particular to the lungs of *Y*. *pestis*-infected mice is correlated with the limited up-regulation of multiple inflammatory cytokines and chemokines. Additionally, pulmonary infection with *Y*. *pestis* creates a permissive environment for the proliferation of other avirulent bacterial species [23].

Bacterial pathogens have developed a variety of mechanisms to inhibit immune cell functions as a means to disarm the host defense. For example, *Y*. *pestis* utilizes the pCD1-encoded type III secretion system (TTSS) composed of a secretory apparatus, chaperones and several translocated effectors (Yops) to disable the early innate immune response [24–26] and the activity of neutrophils in particular [27, 28]. Recently, neutrophils were found to be an important cellular target of *Y*. *pestis* Yop secretion during the early stage of pneumonic plague [29].

While the lack of an adequate early immune response in the lungs during pneumonic plague is well described, the cascade of neutrophil recruitment from the circulation into the lungs during pneumonic plague and the identification of *Y*. *pestis* virulence factors involved in suppressing this process have yet to be fully elucidated.

We previously reported that an early immune response is initiated by bone-marrow (BM) cells after airway infection of mice with a fully virulent *Y*. *pestis* strain, causing rapid mobilization of neutrophils from the BM to the blood circulation [30]. In the present study, we analyzed the interference of *Y*. *pestis* with the recruitment of neutrophils from the circulation to the lungs of infected mice. Our observations indicate that in the lungs of infected mice, the induction of the major neutrophil chemoattractants KC, MIP-2 and G-CSF as well as the leukocytes adhesion molecules E-selectin, P-selectin, ICAM-1 and VCAM-1 is delayed. In addition, we describe the role of YopJ in preventing the induction of the chemoattractants mRNA at the early stage of disease progression. Finally, we demonstrate that early attraction of neutrophils to the infected lung by intranasal installation of exogenous chemoattractants improves bacterial clearance as well as survival rate.

## Results

### Delayed recruitment of neutrophils to the lungs of *Y*. *pestis-*infected mice is associated with rapid bacterial propagation

Studies in animal models of pneumonic plague have revealed the biphasic nature of the progression of this disease. The early phase of the disease, which takes place during the first 24–36 hours post infection (hpi), involves a limited pro-inflammatory response in the lung, whereas the later phase of disease progression (48–72 hpi) is associated with an excessive pro-inflammatory response [20–22].

**Fig 1:**
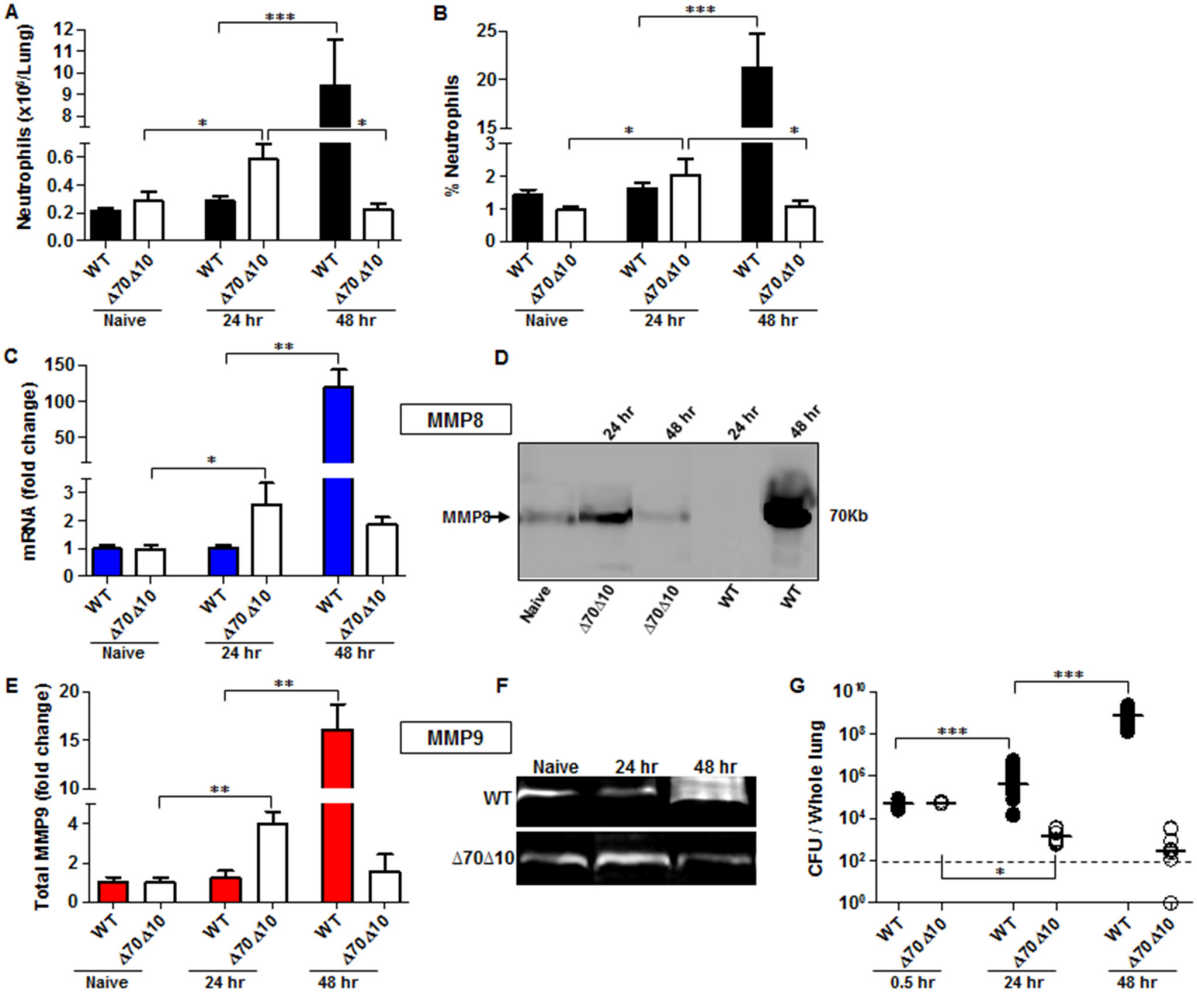
Bacterial propagation and neutrophil infiltration to the lungs following airways infection of mice with *Y*. *pestis* strains. C57BL/6J mice were infected i.n. with 1x10^5^ cfu (100 LD_50_) of the fully virulent *Y*. *pestis* strain Kim53 (WT) or with an equivalent dose of the avirulent mutant strain Kim53Δ70Δ10 (Δ70Δ10) that lacks the pCD1 and pPCP1 plasmids. Cells collected from lung extracts were counted and stained for the neutrophil markers Gr-1 and CD11b and subjected to flow cytometry analysis. Absolute neutrophils number (A) and percentage (B) in the lungs of mice at the indicated time points post i.n. infection with the WT and the Δ70Δ10 strains are presented respectively. The expression levels of MMP8 were determined by qPCR (C) and western blot analysis (D) at the indicated time points after i.n. infection with the WT and Δ70Δ10 *Y*. *pestis* strains. The expression levels of MMP9 were determined by ELISA (E) and Gelatin zymography (F) at the indicated time points after i.n. infection with WT and Δ70Δ10 *Y*. *pestis* strains. MMPs protein and mRNA levels are presented as fold change relative to naïve mice. (G) Bacterial propagation in the lungs of mice infected i.n. with the WT or the Δ70Δ10 strains was determined by plating the samples and calculating the cfu. All results are presented as the means ± SEM (*p<0.05, **p<0.01, ***p<0.001). n >10 mice per group of at least 3 independent experiments.

To further characterize the early innate immune response during pneumonic plague, C57BL/6 mice were exposed i.n. to a lethal dose of 1x10^5^ cfu (100 LD_50_) of the highly virulent *Y*. *pestis* strain Kim53, which typically kills mice within 72 hpi (S1 Fig). As neutrophils are one of the first innate immune cells recruited to the site of infection, we measured the levels of neutrophils infiltrating into the lungs at the early time point of 24 hpi. No significant change was observed in the absolute number or percentage of neutrophils at this time point in comparison to naïve mice (Fig 1A and 1B). Because the metalloproteinases MMP8 and MMP9 are produced and released by neutrophils while combating invading pathogens [31], we measured the levels of MMPs in lung extracts at the early time point of 24 hpi. Consistent with the limited infiltration of neutrophils into the lungs, the expression of MMP8 (Fig 1C and 1D) and MMP9 (Fig 1E and 1F) in the lungs of infected mice did not change in comparison to naïve mice. These findings indicate that the early pulmonary innate response, associated with neutrophils homing to the lung, is impaired after infection with the virulent *Y*. *pestis* strain. Moreover, the increased number of live *Y*. *pestis* bacilli detected in the lungs at 24–48 hpi (Fig 1G) suggests that the delayed influx of neutrophils into the lungs allowed the pathogen to rapidly proliferate and overwhelm the innate immune system. As the disease progressed into the excessive pro-inflammatory state at 48 hpi, massive infiltration of neutrophils into the lungs was observed (Fig 1A and 1B) together with a dramatic increase in the expression of both MMP8 and MMP9 (Fig 1C–1F). Evidently, this late intensive pulmonary immune response was unable to prevent the pathogen from propagating to high levels in the lung tissue (Fig 1G).

This phenomenon was in contrast to the kinetics of neutrophil infiltration after infection with an equivalent infective dose of the avirulent *Y*. *pestis* strain Kim53Δ70Δ10, which lacks the pCD1 and pPCP1 plasmids that carry essential virulence factors including the TTSS and Pla protease (Fig 1A and 1B). The rapid elevation in neutrophil counts in the lungs of mice infected with the avirulent *Y*. *pestis* strain was accompanied by a significant up-regulation of the levels of MMP8 and MMP9 expression (Fig 1C–1F) and by a significant decrease in bacterial loads in the lungs at 24 hpi (Fig 1G). Notably, this early recruitment of neutrophils to the lungs was transient, as depicted by the return of neutrophil numbers in the lungs to their basal level by 48 hpi (Fig 1A and 1B). As a result, the levels of MMP8 and MMP9 expression were also reduced (Fig 1C–1F).

We previously demonstrated that i.n. infection of mice with the virulent *Y*. *pestis* strain is sensed by the BM compartment early after infection, resulting in the subsequent release of neutrophils to the blood by 12–24 hpi [30]. The observed delay in the homing of neutrophils from the circulation to the lungs motivated us to study the impairment of this pathway during the progression of pneumonic plague.

### Expression of chemokines involved in neutrophil chemotaxis to the lungs is delayed during the early stages of pneumonic plague

**Fig 2:**
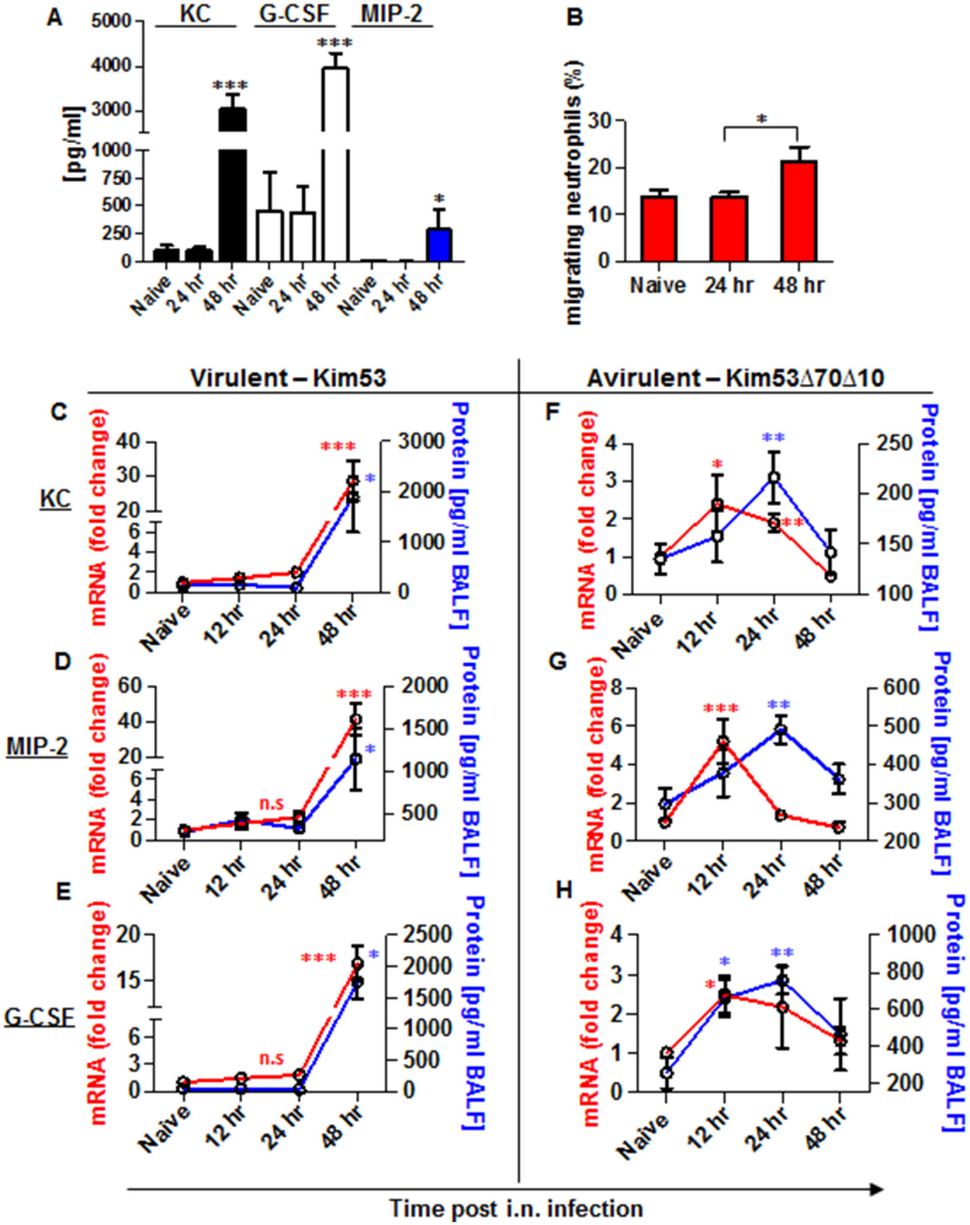
Expression of inflammatory signals involved in neutrophil chemotaxis in the blood and lungs of mice infected i.n. with *Y*. *pestis* strains. C57BL/6 mice were infected i.n. with 1×10^5^ cfu (100 LD_50_) of the virulent *Y*. *pestis* strain Kim53. (A) At the indicated time points post infection, the levels of KC, MIP-2 and G-CSF proteins in the plasma were determined by ELISA. (B) Lung supernatants were extracted from the infected mice at the indicated time points and used for functional Transwell-migration assays with naïve BM-derived neutrophils. (C-E) The levels of KC, MIP-2 and G-CSF protein (blue) and mRNA (red) were determined by ELISA and qPCR analysis in BALF samples and whole lung cells respectively following infection with the virulent *Y*. *pestis* strain Kim53. (F-H) C57BL/6 mice were infected i.n. with 1×10^5^ cfu of the avirulent *Y*. *pestis* strain Kim53Δ70Δ10 and at the indicated time points post infection the protein (blue) and mRNA (red) levels of KC, MIP-2 and G-CSF were determined. All results are presented as the means ± SEM (*p<0.05, **p<0.01, ***p<0.001). Protein and mRNA levels are presented as fold change relative to naïve mice. n >10 mice per group of at least 3 independent experiments.

Because the chemoattractants KC, MIP-2 and G-CSF are of central importance for the recruitment of neutrophils to infected organs [32], their levels in the blood of mice infected with the virulent *Y*. *pestis* strain were measured by ELISA. As shown, the levels of KC, MIP-2 and G-CSF at the early stage of 24 hpi were comparable to those observed in naïve mice, and these levels increased significantly only during the late stage of infection at 48 hpi (Fig 2A). This result suggests that although neutrophils are released from the BM to the blood early after airway infection, their ability to navigate towards the infected lungs is impaired. To better understand the limited ability of infected lungs to induce neutrophil chemotaxis and infiltration, we performed a Transwell-migration assay of naïve BM-derived neutrophils towards lung supernatants obtained from mice at several time points after infection with *Y*. *pestis* Kim53. Only lung supernatants obtained from mice at late stages of disease progression e.g., 48 hpi, demonstrated the potential to induce *in vitro* Transwell-migration of naïve neutrophils (Fig 2B), implying that at this time point, the lungs are enriched with chemotactic factors that facilitate neutrophil migration.

Next, we measured the levels of mRNA and protein expression of KC, MIP-2 and G-CSF in lung extracts and BALF at 12, 24 and 48 hpi with Kim53. The levels of mRNA (red line) and protein (blue line) of KC, MIP-2 and G-CSF were significantly increased in the lungs only at 48 hpi (Fig 2C–2E). These observations are in line with previous reports describing the delayed pulmonary pro-inflammatory response to *Y*. *pestis* infection using various animal models of pneumonic plague [20–22, 33].

In contrast, infection with the avirulent *Y*. *pestis* strain Kim53Δ70Δ10 was characterized by early and moderate induction of the mRNA levels of KC, MIP-2 and G-CSF in the lungs at 12 hpi, accompanied by elevated protein levels in the BALF (Fig 2F and 2H).

### Expression of adhesion molecules in the lungs is delayed during the early stages of pneumonic plague

**Fig 3:**
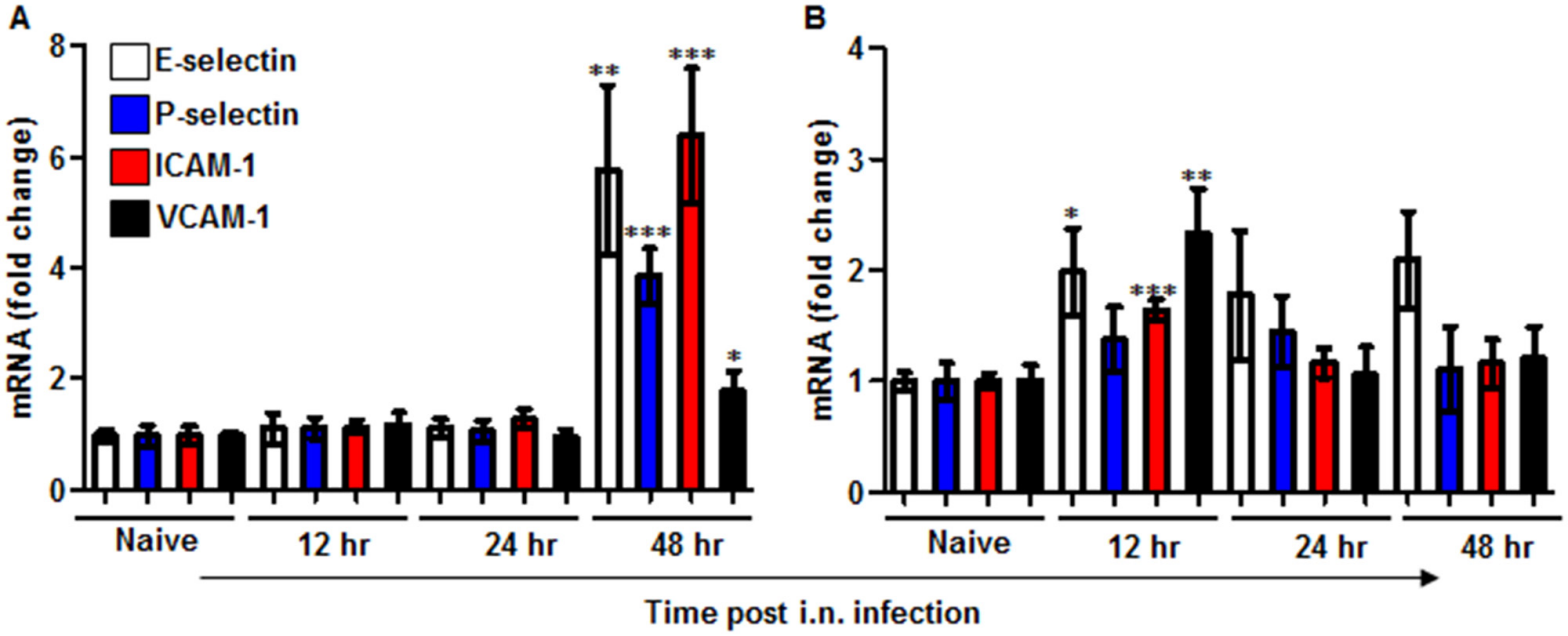
Expression of adhesion molecules in the lungs of mice infected i.n. with *Y*. *pestis* strains. C57BL/6 mice were infected i.n. with 1×10^5^ cfu (100 LD_50_) of the virulent *Y*. *pestis* strain Kim53 (A) or the same dose of the avirulent *Y*. *pestis* Kim53Δ70Δ10 (B). At the indicated time points post infection, mRNA was purified from the infected lungs and subjected to qPCR analysis of E-selectin, P-selectin, ICAM-1 and VCAM-1 gene expression. The results are presented as the means ± SEM (*p<0.05, **p<0.01, ***p<0.001). mRNA levels are presented as fold change relative to naïve mice.

The recruitment of neutrophils to infected tissue is a complex process dependent on orchestrated and tightly regulated communication between neutrophils and endothelial cells, resulting in a multistep adhesion cascade. This process includes the initial attachment of the neutrophils to the endothelium, rolling along the endothelial surface and arrest at the final destination to allow complete transmigration [34]. This sequence of coordinated and transient interactions relies on the synchronized expression of several adhesion molecules by endothelial cells adjacent to the site of inflammation. Hence, we decided to examine the expression levels of four molecules that participate in two different stages of neutrophil transmigration: E-selectin, P-selectin, ICAM-1 and VCAM-1 [7]. Quantitative PCR analysis of lung mRNA obtained from mice infected with the virulent Kim53 strain revealed a delay in the up-regulation of the expression of all four adhesion molecules during the first 24 hpi (Fig 3A). Again, this delayed expression was in contrast to the early induction of adhesion molecule mRNA in the lungs of mice infected with the avirulent strain Kim53Δ70Δ10 (Fig 3B). Together, these data suggest that the rapid propagation of *Y*. *pestis* in the lungs during the early stages of pneumonic plague results from an impaired innate immune response associated with delayed neutrophil infiltration to the lung, presumably due to a combined delay in the expression of chemotactic signals and adhesion molecules by lung resident cells.

### YopJ is involved in suppressing the induction of G-CSF, KC and MIP-2 following *in vitro* infection of lung-derived cell lines with *Y*. *pestis*

**Fig 4:**
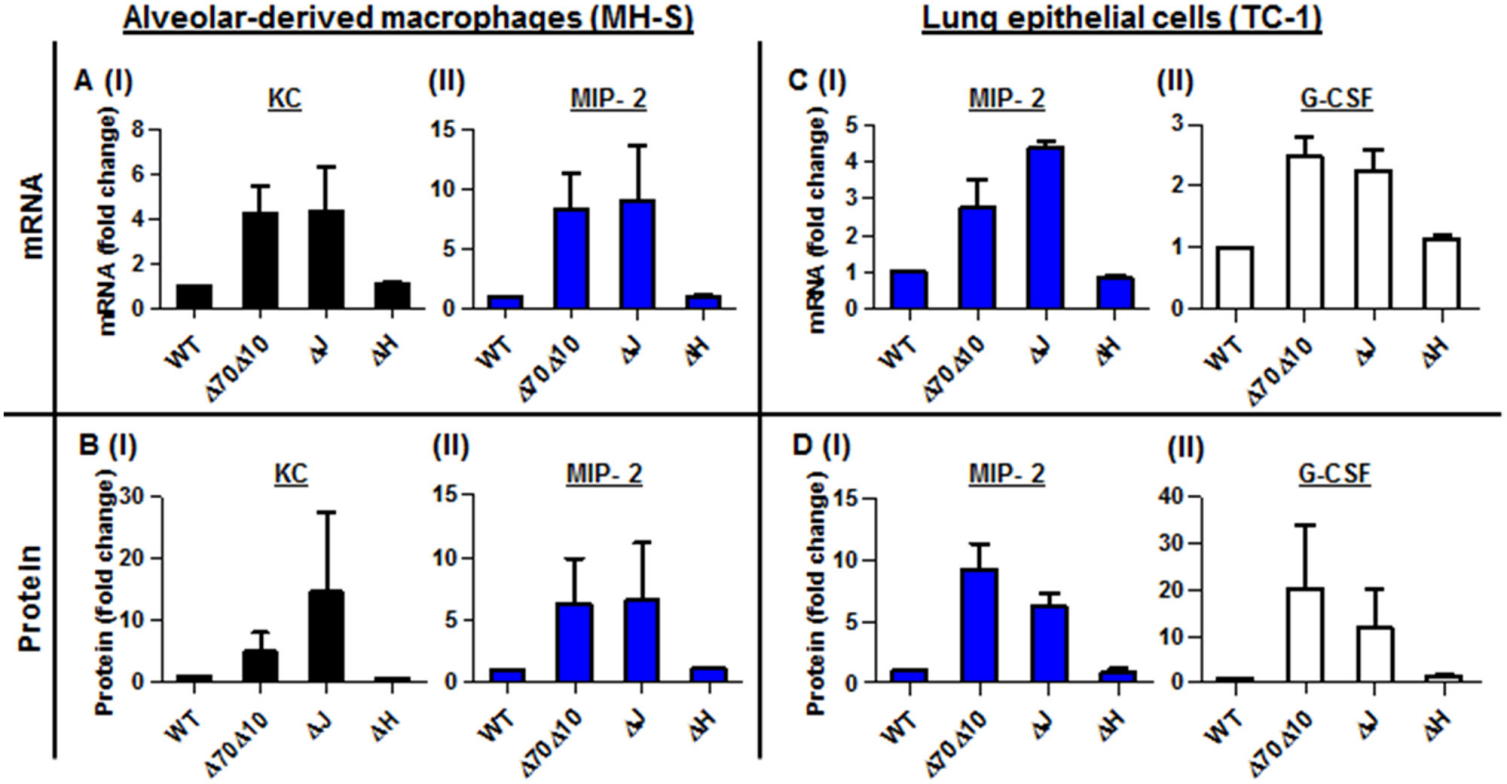
*In vitro* infection of alveolar macrophages and lung epithelial cells with *Y*. *pestis* strains. *In vitro* infection of MH-S alveolar-derived macrophages (A-B), and TC-1 lung-derived epithelial cell lines (C-D) with 50 MOI of the virulent *Y*. *pestis* strain Kim53 (WT) and its Yop-depleted derivatives Kim53ΔYopJ (ΔJ), Kim53ΔYopH (ΔH) and Kim53Δ70Δ10 (Δ70Δ10). The mRNA (A and C) and protein (B and D) levels were quantified using qPCR and ELISA, and are presented as the fold change relative to the WT-Kim53 *Y*. *pestis* strain.

The virulence characteristics of *Y*. *pestis* are mostly attributed to the pCD1 plasmid that encodes the TTSS and its six effectors proteins, termed Yops. During interactions with host target cells, these proteins are transported into the cytosol of the host cell via a needle-like apparatus. Together, the translocated Yops target the phagocytic machinery and deregulate signaling pathways, resulting in a reduced immune response by the host [35–37]. As described, infection of mice with the avirulent strain Kim53Δ70Δ10 that does not express the entire TTSS and the Pla protease, was characterized by the early induction of neutrophil chemoattractant mRNA and protein in the lungs (Fig 2F and 2H), and by the early recruitment of neutrophils to the lungs (Fig 1A and 1B). We suspected that one of the Yop effectors may be involved in the early suppression of the up-regulation of chemoattractants expression in the lungs after exposure to the virulent *Y*. *pestis* strain. To decipher which Yop was responsible for the early inhibition of KC, MIP-2 and G-CSF up-regulation, we used two different Yop-null derivatives of the fully virulent *Y*. *pestis* strain Kim53, and we performed a series of *in vitro* infection experiments using alveolar-derived macrophages (MH-S) and lung epithelial (TC-1) cell lines. We used the avirulent *Y*. *pestis* strain Kim53Δ70Δ10 and the wild-type *Y*. *pestis* strains as controls in these experiments. As shown, infection with 50 MOI of the Kim53ΔYopJ strain, but not with the Kim53 derivative lacking YopH, resulted in increased levels of KC and MIP-2 mRNA and protein in MH-S cells (Fig 4A and 4B) and of MIP-2 and G-CSF mRNA and protein in TC-1 cells (Fig 4C and 4D). The induction of these chemokines production by the cell lines following infection with the YopJ mutant was similar to infection with the avirulent strain Kim53Δ70Δ10. Additionally, infection of both cell lines with a YopJ mutant of the *Y*. *pestis* EV76 vaccine strain and with an EV76 derivative lacking the pCD1 plasmid led to induction of chemoattractants mRNA, whereas infection with EV76 and other Yop-null mutants including EV76ΔYopE, EV76ΔYopK and EV76ΔYopH did not (S2 Fig). These results point to the involvement of YopJ in the regulation of KC, MIP-2 and G-CSF expression by lung-derived cells *in vitro*.

### The virulence of the Kim53 YopJ-depleted mutant is associated with inhibition of KC, MIP-2 and G-CSF protein but not mRNA expression in the lung

**Fig 5:**
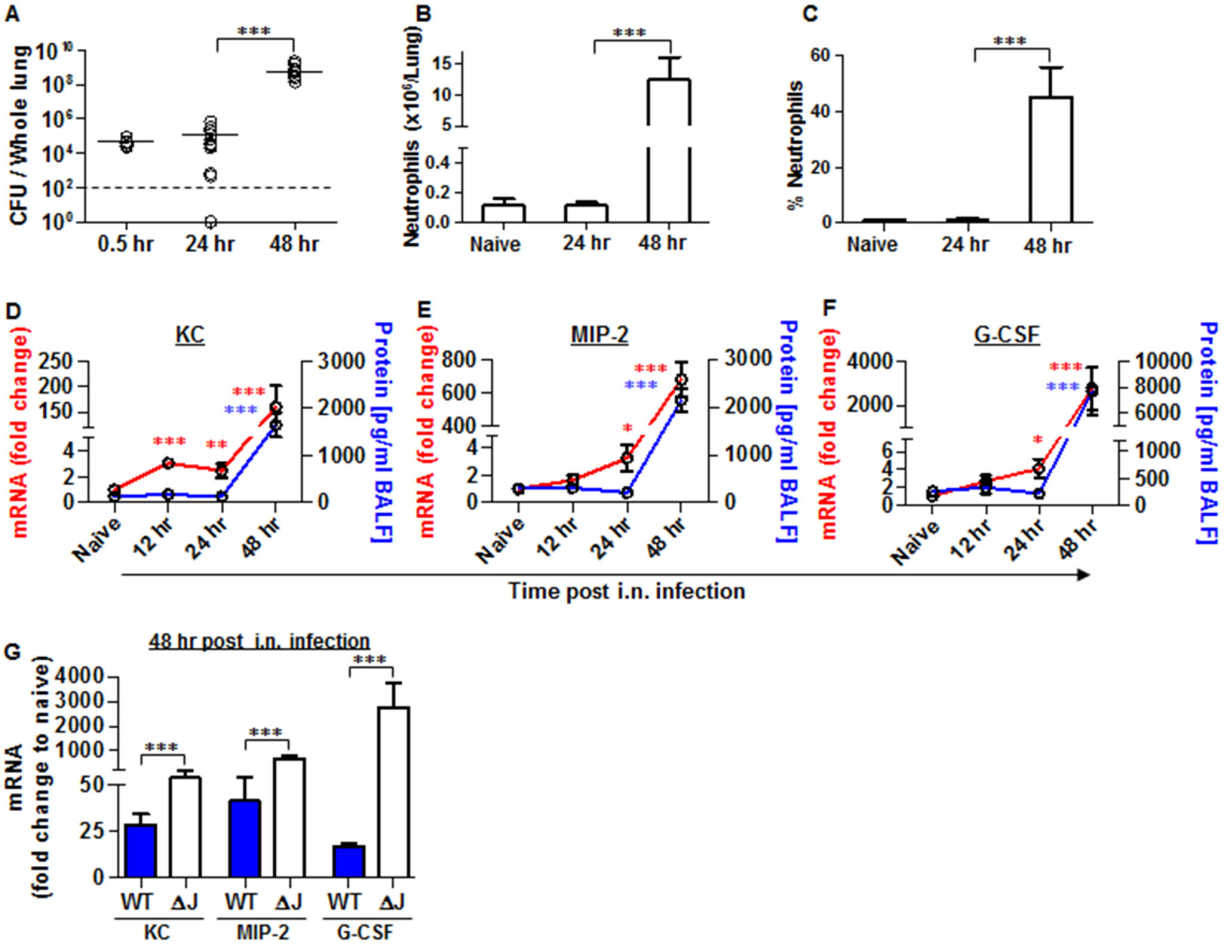
Neutrophils homing to lungs and bacterial propagation following airway infection of mice with the YopJ-deleted strain (Kim53ΔYopJ). Bacterial propagation in the lungs of C57BL/6J mice infected i.n. with 1x10^5^ cfu of the mutated *Y*. *pestis* strain Kim53ΔYopJ was determined by plating the samples and calculating the cfu. Absolute numbers (B) and percentages (C) of neutrophils in the lungs of the infected mice at the indicated time points post infection were determined by flow cytometry analysis as described in Fig 1. Whole lung extracts or BALF were collected at the indicated time points post i.n. infection with 1x10^5^ cfu of *Y*. *pestis* strain Kim53ΔYopJ and then analyzed for the mRNA expression (red line) and protein level (blue line) of KC (D), MIP-2 (E) and G-CSF (F). (G) Comparison between the mRNA levels of KC, MIP-2 and G-CSF in the lungs of C57BL/6J mice at 48 hr post i.n. infection with 1x10^5^ cfu of Kim53 (WT) or Kim53ΔYopJ (ΔJ). Fold changes were determined in comparison to naïve mice. The results are presented as the means ± SEM (*p<0.05, **p<0.01, ***p<0.001). n = 6–12 mice per group of at least 3 independent experiments.

Because YopJ activity was associated with suppression of KC, MIP-2 and G-CSF expression in *Y*. *pestis*-infected alveolar macrophages and epithelial cells, we infected C57BL/6 mice i.n. with a dose of 1x10^5^ cfu of Kim53ΔYopJ and monitored the disease progression in the lung. All infected mice succumbed within 4 days of infection (S1 Fig). Bacterial counts in the lungs were elevated to 1x10^9^ cfu by 48 hpi (Fig 5A), and no significant change was observed in neutrophil numbers or percentage in the lungs after the first 24 hpi (Fig 5B and 5C). Massive infiltration of neutrophils to the lungs was apparent only at 48 hpi (Fig 5B and 5C), and the delayed kinetics of neutrophil influx to the lungs following i.n. infection with Kim53ΔYopJ and bacterial propagation resembled the kinetic responses observed following infection with the wild-type Kim53 strain (Fig 1). Taking into account the observed involvement of YopJ in preventing KC, MIP-2 and G-CSF up-regulation by lung resident cells *in vitro* (Fig 4), we further evaluated the changes in mRNA and protein levels of these chemokines in lung extracts and BALF of Kim53ΔYopJ-infected mice. Surprisingly, we observed that while the mRNA levels of these chemokines were significantly elevated at 12–24 hpi, their protein levels remained constant and relatively low during this time frame. A significant increase in the protein levels of G-CSF, KC and MIP-2 was detected only at 48 hpi with Kim53ΔYopJ (Fig 5D and 5F). Notably, significantly higher levels of G-CSF, KC and MIP-2 mRNA were measured at 48 hpi in the lungs of mice infected with the YopJ mutant compared to those of mice infected with the wild-type *Y*. *pestis* strain (Fig 5G). This difference may result from earlier induction of chemoattractants mRNA following infection with the YopJ mutant, leading to the accumulation of higher levels of chemoattractants mRNA at later stages of disease progression. The data indicate that YopJ mediates the delayed up-regulation of chemoattractant mRNA expression in the lungs of *Y*. *pestis*-infected mice. However, unlike in the *in vitro* infection system we utilized, in the absence of YopJ, the early elevation of chemoattractant mRNA levels did not yield a subsequent increase in the protein levels. These observations suggests that additional virulence mechanisms involving host-pathogen interactions play a role in modulating the early expression of chemoattractants in a complex multicellular organ such as the lungs, thereby preventing the early recruitment of neutrophils to the infected lung.

### Intranasal instillation of recombinant KC and MIP-2 along with systemic administration of G-CSF (GKM), induce the early recruitment of neutrophils to the lungs during pneumonic plague

**Fig 6:**
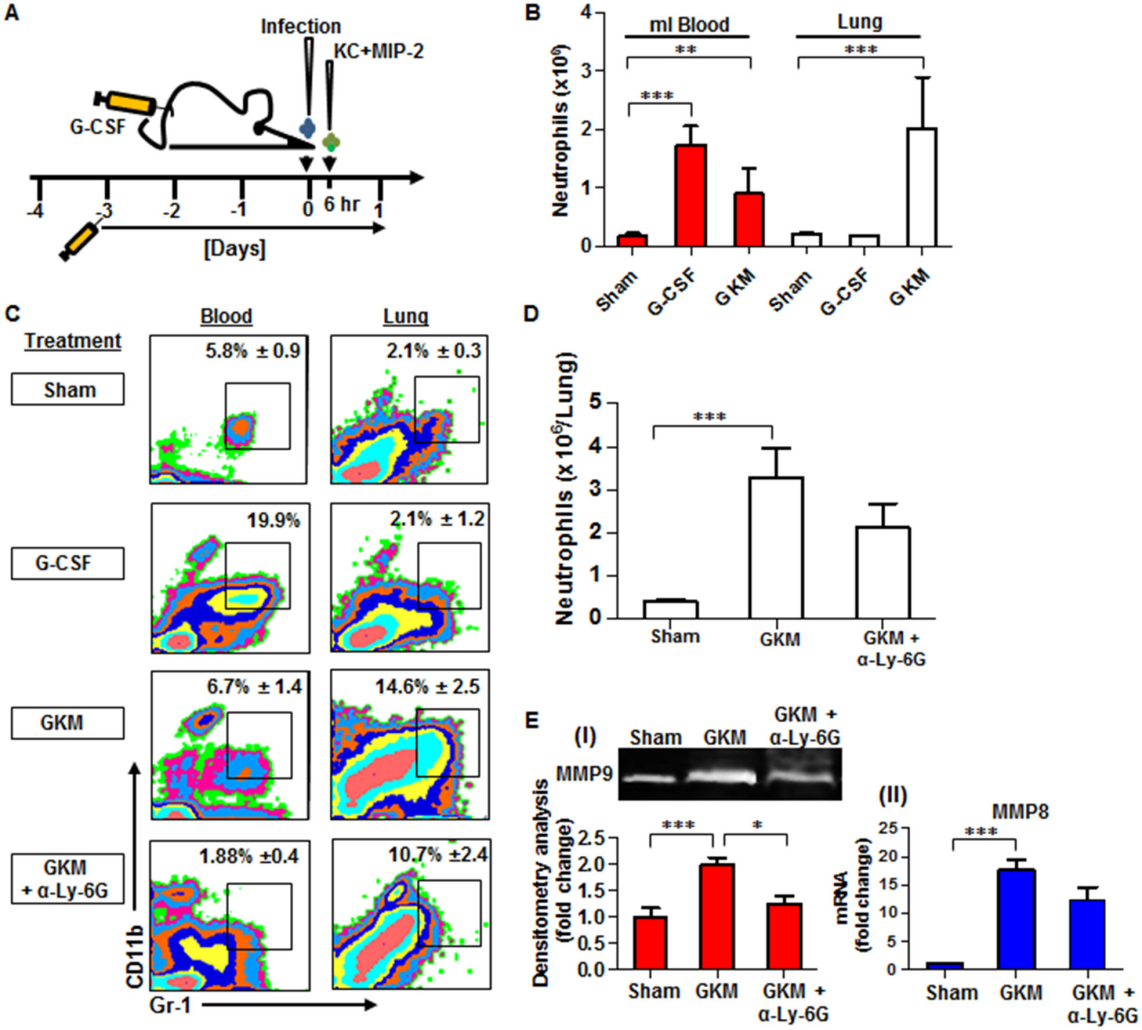
Combined treatment with G-CSF, KC and MIP-2 (GKM) facilitates the early recruitment of neutrophils to the lungs during pneumonic plague. (A) Schematic presentation of the GKM treatment regimen. Mice were treated for 5 consecutive days with a daily subcutaneous injection of G-CSF, starting 3 days prior to i.n. infection with 1×10^5^ cfu (100LD_50_) of the virulent *Y*. *pestis* strain (Kim53). Six hours after infection, recombinant KC and MIP-2 proteins (1 μg/mouse each) were administered i.n., and mice were sacrificed at 24 hpi. (B) Neutrophil numbers (determined by flow cytometry analysis), in the blood and lungs of naïve mice treated with: PBS (sham), G-CSF alone or the full GKM treatment as described above. (C) Representative flow cytometry analysis of the percentages of neutrophils determined in the blood and lungs of *Y*. *pestis*-infected mice at 24 hours post i.n. infection with 1×10^5^ of the Kim53 strain. The percentages of neutrophils are indicated in non-treated mice compared with mice treated with G-CSF alone, GKM or GKM together with the neutralizing antibody α-Ly-6G. (D) Absolute neutrophil numbers in the lungs of mice at 24 hr after i.n. infection with 100 LD_50_ of Kim53, treated or not with GKM or GKM+αLy-6G. (E) Expression of MMP9 in whole lung supernatant (I) and expression of MMP8 mRNA in whole lung extracts (II) at 24 hpi with 100LD_50_ of Kim53. Mice were treated as described above with PBS (sham), GKM or GKM+αLy-6G. All results are presented as the means ± SEM (*p<0.05, **p<0.01, ***p<0.001). n >10 mice per group of at least 3 independent experiments.

The early recruitment of neutrophils to the lungs appears to be of central importance for the defense against pneumonic plague. Due to the fact that some key players in this process (e.g. KC, MIP-2 and G-CSF) are targeted by the bacterium early after the infection, we tested the potential of chemokine therapy for early neutrophil recruitment to the lung. The treatment included subcutaneous administration of G-CSF to synchronize and overload the circulation with newly formed neutrophils, combined with i.n. instillation of KC and MIP-2 to guide the neutrophils and stimulate their recruitment and homing to the infected lungs (Fig 6A).

We first examined the potential of this treatment regimen to promote neutrophil recruitment to the lungs of naïve mice. As depicted in Fig 6B, treatment with G-CSF alone for 5 consecutive days significantly increased the numbers of neutrophils in the blood but not in the lung, whereas the combined treatment (GKM), which included an additional intranasal administration of KC and MIP-2 (1 μg/mouse each), led to a significant accumulation of neutrophils in the lungs (Fig 6B). This treatment was not associated with deleterious effects on animal morbidity or mortality (S1 Fig). Next, we assessed the ability of GKM treatment to induce the early recruitment of neutrophils to the lungs of mice infected i.n. with the virulent *Y*. *pestis* strain.

Similar to naïve mice, the percentage of neutrophils measured at 24 hpi in the blood of *Y*. *pestis*-infected mice treated for 5 consecutive days with G-CSF alone (starting 3 days before the infection) was elevated in comparison to the percentage in control-treated mice, whereas the percentage of lung neutrophils did not change (Fig 6C, G-CSF). In contrast, a rapid increase in the percentage and total number of neutrophils was detected at 24 hpi in the lungs of GKM-treated mice that received KC and MIP-2 at 6 hpi (Fig 6C and 6D, GKM). Injection of the specific anti-neutrophil antibody anti-Ly-6G into GKM-treated mice resulted in a reduction in the influx of neutrophils to the lungs by nearly 30%, verifying the specificity of the response with regard to the involvement of neutrophils (Fig 6C and 6D, GKM+αLy-6G). We further assessed whether the early influx of neutrophils to the lungs of GKM-treated mice led to induction of the MMP8 and MMP9 metalloproteinases. Indeed, their expression was significantly higher in lung extracts obtained from GKM-treated mice as compared to control-treated mice (Fig 6E). Again, injection of anti-Ly-6G to GKM-treated mice lowered the expression of MMPs (Fig 6E), indicating that these early recruited neutrophils are in an active state once they reach the lung.

### Early recruitment of neutrophils is associated with clearance of *Y*. *pestis* from the lungs and improved survival rate

**Fig 7:**
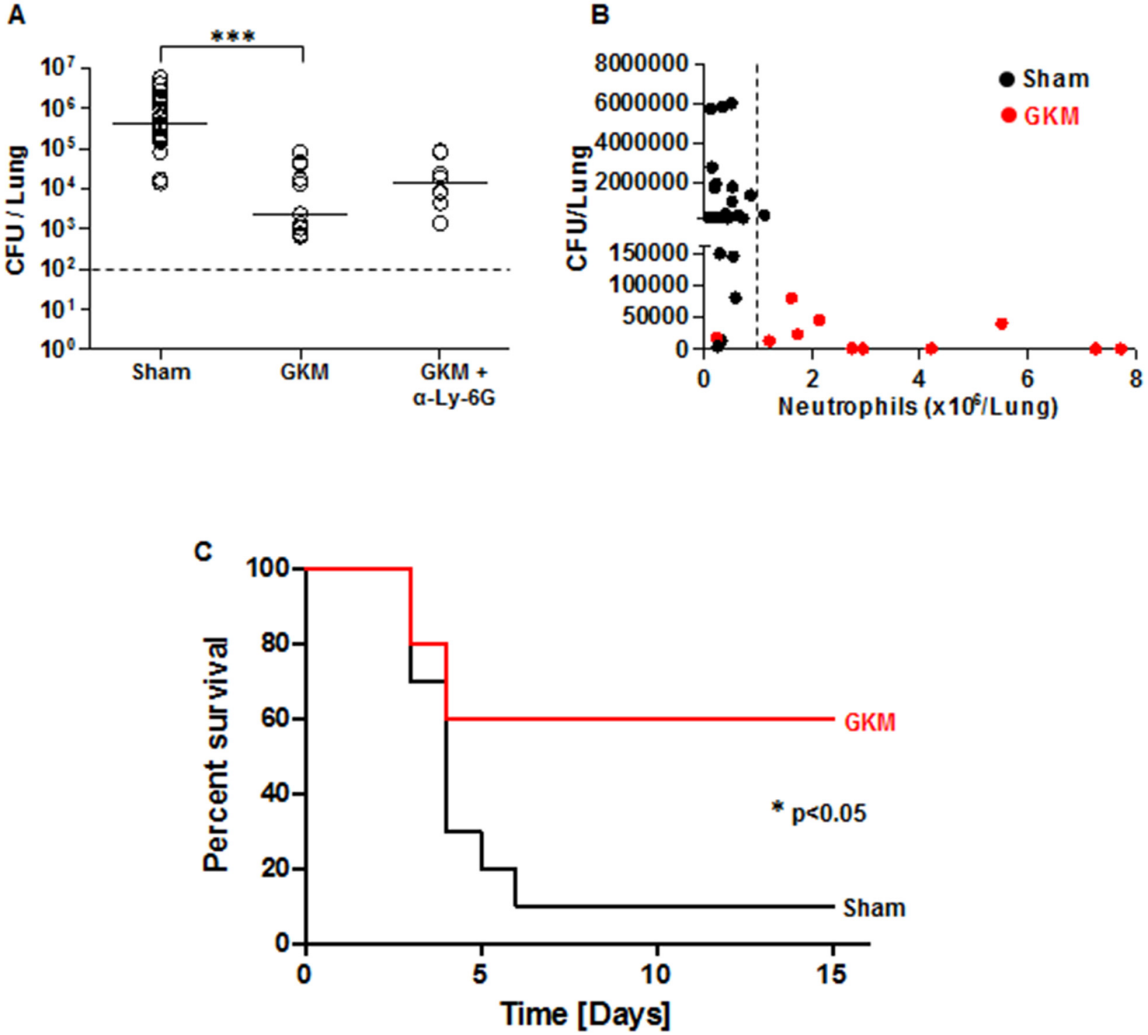
Early recruitment of neutrophils to the lungs is accompanied by reduced bacterial load and improved survival. (A) The bacterial load in the lungs of mice was measured at 24 hr after i.n. infection with 100 LD_50_ of Kim53. Mice were treated with GKM or GKM+αLy-6G and compared to mice receiving the sham treatment (PBS) as described in Fig 6A. The results of cfu counting in the lungs are presented. (B) Correlation between neutrophil level in the lungs and bacterial load at 24 hr following i.n. infection. Black dots represent sham-treated mice, whereas red dots represent GKM-treated animals. The dashed line indicates the threshold of neutrophil numbers in the lung, which was inversely correlated with the bacterial burden. n>8 mice per group of at least 3 independent experiments. (C) Survival of mice treated with PBS (sham) or with GKM after i.n. challenge with 2 LD_50_ of Kim53 (n = 10 mice per group from 2 independent experiments). The results are presented as the means ± SEM (*p<0.05, ***p<0.001).

To determine whether early recruited neutrophils to the lungs are able to clear *Y*. *pestis*, we measured the bacterial burden in the lungs of GKM-treated mice at 24 hr following infection with 1x10^5^ cfu (100LD_50_) of the virulent Kim53 strain. A substantial reduction of almost a thousand fold was observed for the load of *Y*. *pestis* in the lung in comparison to untreated mice (Fig 7A). This beneficial effect of GKM treatment was mediated by neutrophils, as the injection of anti-Ly-6G neutralizing antibody to GKM-treated mice decreased bacterial clearance, reflecting the importance of neutrophils for lung defense against *Y*. *pestis* (Fig 7A). Furthermore, analysis of the relationships between the numbers of neutrophils and bacterial loads in the lungs of GKM-treated versus sham-treated mice at the early time point of 24 hpi, indicated that under this treatment levels of neutrophils greater than 1–2×10^6^ were associated with effective bacterial clearance (Fig 7B).

Following the demonstration of early migration of neutrophils to the lung by the treatment with the recombinant proteins and the pronounced effect on *Y*. *pestis* propagation, it was interesting to evaluate this treatment in animals exposed to a lethal challenge. Relatively high protection level of 60% was observed in GKM-treated mice that were exposed i.n. to a dose of 2x10^3^ cfu of the virulent Kim53 strain, whereas only 10% of the sham-treated mice survived this infection (Fig 7C).

## Discussion

The ability of bacterial pathogens to prevent the early recruitment of neutrophils to infected organs provides an obvious advantage during infection because these cells, with their various antimicrobial capabilities, would otherwise kill the pathogen. *Y*. *pestis*, the causative agent of plague, exploits a variety of mechanisms for evading and coping with the host immune response during the early stages of infection. Accumulating evidence based on studies in various animal models of pneumonic plague indicates that following airway infection with *Y*. *pestis*, the early induction of a pro-inflammatory immune response in the lungs as well as the recruitment of neutrophils to the lungs are delayed [20–22].

We previously showed that the immune response is initiated by BM cells early after i.n. infection of mice with a fully virulent *Y*. *pestis* strain, causing rapid modulation of the BM CXCR4-SDF-1 axis and prompt mobilization of neutrophils into the circulation within 12–24 hpi [30]. These observations raised intriguing questions, namely, at what stage of neutrophil recruitment to the lungs does the pathogen interfere and which *Y*. *pestis* virulence factor is involved in this process. In this study, we further analyzed the mechanisms involved in the late homing of neutrophils to the lungs following i.n. infection of mice with the fully virulent *Y*. *pestis* strain Kim53.

Our results clearly indicate that the influx of neutrophils to the lungs in *Y*. *pestis*-infected mice is delayed. In addition, the delayed recruitment of neutrophils to the lungs is associated with a significant increase in bacterial burden. Cytokines and chemokines act in a coordinated manner to mobilize and recruit neutrophils to the site of inflammation. Because production of these factors represents the first step in the neutrophil recruitment process, we monitored the expression of several chemokines critical for the chemoattraction of neutrophils, in the lungs and plasma of infected mice during disease progression. Up-regulation of G-CSF, KC and MIP-2 in the plasma of infected mice was delayed until the late stages of disease progression (i.e., 48 hpi), consistent with the absence of a pro-inflammatory response in the lungs at the early stage of disease progression [20–22]. In addition, the levels of CXCR2 (KC and MIP-2 receptor), did not change at the first 24 hpi on circulating neutrophils in the blood (S3 Fig).

Using a Transwell-migration assay, we found that lung extracts from the early stage of disease progression were incapable of inducing neutrophil migration, in contrast to lung extracts obtained from mice at later stages of the disease. This result suggests that the intrinsic induction of chemoattractant production in lung resident cells early after *Y*. *pestis* infection is inhibited. Moreover, the delayed induction of KC, MIP-2 and G-CSF mRNA and protein in the lungs of Kim53-infected mice corroborates this observation.

The expression of adhesion molecules is up-regulated on endothelial cells located at the site of inflammation [38]. Circulating neutrophils that egress from the BM undergo E- and P-selectin-mediated rolling along the endothelial surface, followed by firm attachment via ICAM-1 and VCAM-1 [39]. In addition to the delay in chemokine up-regulation, the ability of Kim53-infected lung cells to support neutrophil transmigration into the lungs also appears to be impaired at the early stage of infection. This is due to the delayed induction of the adhesion molecules E- and P-selectin as well as ICAM-1 and VCAM-1. Because chemokines are involved in the expression of adhesion molecules on capillary endothelia [1], the delayed induction of adhesion molecules in *Y*. *pestis*-infected lungs might result from delayed expression of the KC and MIP-2 chemokines. Alternatively, direct interaction of *Y*. *pestis* with endothelial cells might affect the expression of these molecules, as demonstrated for ICAM-1 during *in vitro* infection of human umbilical vein endothelial cells (HUVECs) with the related enteropathogen *Y*. *enterocolitica* [40].

The virulence of pathogenic *Yersinia* strains is mostly attributed to the TTSS and its effector proteins which are used by the pathogen to subvert early innate immune responses [26, 41]. In striking contrast to the impaired innate immune response in the lungs of mice infected i.n. with the virulent *Y*. *pestis* strain Kim53, rapid and moderate induction of the expression of chemokines and adhesion molecules followed by an influx of neutrophils to the lungs was observed early after pulmonary infection of mice with the avirulent *Y*. *pestis* strain (Kim53Δ70Δ10) that lacks the TTSS and Pla protease virulence factors. Consequently, this prompt response was associated with effective bacterial clearance from the lungs.

TTSS Yop effectors of pathogenic *Yersinia* species are known for their ability to suppress the induction of innate immune responses in various types of mammalian cells through disruption of the target cell signaling network. For example, YopE has been reported to inhibit the production of IL-8 (the human homologue of KC) in *Y*. *pseudotuberculosis-*infected HeLa cells [42, 43], and YopJ-dependent suppression of TNFα secretion has been reported in *Yersinia*-infected macrophages [44–46]. Human bronchial epithelial cells co-transfected with cDNAs encoding *Y*. *pseudotuberculosis* YopJ also exhibited reduced transcription of IL8, RANTES and ICAM-1 in a promoter activity assay [47]. Moreover, the YopH effector was shown to inhibit the expression of monocyte chemoattractant protein 1 (MCP-1) in macrophages infected with *Y*. *enterocolitica* [48] and to suppress early pro-inflammatory cytokines in the lungs during pneumonic plague [49].

We investigated the involvement of YopJ and YopH in the modulation of KC, MIP-2 and G-CSF expression by alveolar macrophages (MH-S) and lung epithelial cell lines (TC-1) following infection of the cells with *Y*. *pestis* Kim53 YopJ and YopH deficient mutants. The *in vitro* results indicated that inhibition of KC, MIP-2 and G-CSF mRNA and protein expression was mediated by the YopJ effector in a similar manner that was shown after infection with the avirulent *Y*. *pestis* strain—Kim53Δ70Δ10, which lacks the entire TTSS.

YopJ (YopP in *Y*. *enterocolitica*) belongs to a family of proteases related to the ubiquitin-like protein proteases [50], and YopJ was shown to be a deubiquitinating cysteine protease capable of removing ubiquitin moieties from IκBα, thereby inhibiting its proteasomal degradation and leading to the down-regulation of NF-κB function [51]. In addition, YopJ was shown to acetylate Ser/Thr residues in the activation loop of MAPK kinases (MKKs) and IκB kinases (IKKs), thereby preventing their activation by phosphorylation [52, 53]. This acetyltransferase activity of YopJ may well account for its ability to inhibit MAPK pathways and NF-κB activation. Interestingly, expression of the KC and MIP-2 genes is known to be tightly regulated by the NF-κB transcription factor [54, 55], and NF-κB activation in lung epithelial cells was shown to be important for the migration of neutrophils to the lungs [56]. Therefore, YopJ-mediated inhibition of NF-κB activity may be involved in the suppression of KC and MIP-2 expression in lung-derived cell lines infected with *Y*. *pestis*.

Studies on the involvement of YopJ in *Y*. *pestis* virulence have indicated that this effector is not essential for virulence in various rodent models of plague [57–59]. Similar results were obtained in our study using C57BL/6 mice infected i.n. with the Kim53ΔYopJ mutant. Close examination of the expression of neutrophil chemoattractants in the lungs of Kim53ΔYopJ-infected mice revealed that while the mRNA levels of KC, MIP-2 and G-CSF were induced in the lungs early after infection, the proteins levels were up-regulated only at the late stage of the disease, *i*.*e*., 48 hpi. In line, the influx and homing of neutrophils to the lungs of Kim53ΔYopJ-infected mice was also delayed to late stage of the disease as observed following exposure to the wild-type *Y*. *pestis* strain. These intriguing data may indicate that *Y*. *pestis* affects the early recruitment of neutrophils to the lungs by regulating the expression of neutrophil chemoattractants at both the transcriptional level via the YopJ effector and at the posttranscriptional level by another, yet unknown virulence factor. One possible candidate is the YopH tyrosine phosphatase that was shown to suppress early pro-inflammatory cytokine up-regulation in the lungs of *Y*. *pestis*-infected mice; this factor was also shown to be essential for *Y*. *pestis* virulence in the mouse model of pneumonic plague [49]. Another candidate is the Pla protease that was shown to be important for *Y*. *pestis* proliferation in the lungs [60] and for degradation of Fas ligand to manipulate host cell death and inflammation [61]. The apparent discrepancy between the *in vitro* and *in vivo* infection systems suggests a different mode of chemokines expression in the absence of YopJ. While YopJ depletion resulted in augmented mRNA and protein expression levels of KC, MIP-2 and G-CSF by infected macrophages and epithelial cell lines *in vitro*, lungs obtained from infected mice exhibited changes only in the mRNA and not in the protein levels of these chemokines early after infection. We assume that these differences emphasize the major impact of the alveolar niche and its various resident cells on the complex virulent mechanisms generated by *Y*. *pestis*. Furthermore, the lack of host defense components (such as other white blood cells, immunoglobulins, complement, cytokines, defensins and more) in *in vitro* models, may have a great influence on the virulence quality of the pathogen.

Because we previously showed that neutrophils egressed from the BM at 12–24 hr after i.n. infection with *Y*. *pestis* [30], our current findings point to the delayed induction of chemokines as the major reason for the inability of circulating neutrophils to rapidly infiltrate the site of infection in the lungs. To test this hypothesis, we administered recombinant KC and MIP-2 by i.n. instillation into the lungs of *Y*. *pestis*-infected mice that were pre-treated systemically with G-CSF to synchronize and overload the circulation with primed neutrophils [62, 63].

Whereas systemic pre-treatment of mice with G-CSF alone prior to pulmonary infection with *Y*. *pestis* did not lead to early recruitment of neutrophils to the lungs, additional i.n. administration of KC and MIP-2 several hours after the infection resulted in rapid mobilization of neutrophils to the lungs. Furthermore, the recruitment of neutrophils to the lungs of treated mice was accompanied by an increase in neutrophil-associated MMP8 and MMP9 expression and induction of the expression of the adhesion molecules E- and P-selectin in the lungs (S4 Fig).

The early influx of neutrophils to the lungs of *Y*. *pestis*-infected mice led to a rapid and significant reduction of nearly 1,000-fold in the average bacterial cfu in the lungs of mice infected with a dose of 100 LD_50_ of the virulent *Y*. *pestis* Kim53 strain. Moreover, this treatment was also successful in improving the survival rates of mice following i.n. exposure to a lethal dose of 2 LD_50_ of the fully virulent *Y*. *pestis* strain. Injection of treated mice with neutralizing anti-Ly-6G antibodies reduced the percentages of neutrophils by 30% and diminished the beneficial antibacterial outcome of treatment as well as the expression of MMPs, highlighting the contribution of recruited neutrophils to lung defense against *Y*. *pestis*.

Collectively, our results indicate that *Y*. *pestis*-mediated interference with the early induction of G-CSF, KC and MIP-2 expression by lung resident cells and the recruitment of neutrophils to the lungs are important for the manifestation of *Y*. *pestis* virulence during pneumonic plague. These observations are consistent with previous reports demonstrating (a) the delayed expression of pro-inflammatory cytokines and chemokines during pneumonic plague [20–22] and (b) the importance of neutrophils for defense against *Y*. *pestis* pulmonary infection [15]. Notably, Goldman and his colleagues have recently reported that the number of neutrophils in the lungs of *Y*. *pestis*-infected mice increases rapidly within 24 hr post intranasal infection [29]. Differences in the experimental systems may account for this discrepancy, as this group infected the mice with an inoculation dose of 10^6^ cfu of the CO92 strain pre-grown at 37°C, whereas in our experiments mice were infected with a lower dose of 10^5^ cfu of the Kim53 strain pre-grown at 28°C.

Directed guidance of immune cells to the lungs following i.n. administration of recombinant proteins has become an important tool for understanding the lung defense mechanisms against bacterial infections. Intranasal administration of exogenous KC ameliorated *B*. *pertussis-*mediated inhibition of neutrophil recruitment to the lungs in infected mice [64]. In addition, the recruitment of neutrophils to the lungs of *S*. *pneumonia*-infected mice was observed following i.n. administration of IL-12 and was associated with increased levels of KC, decreased bacterial burden and improved survival [65]. The early arrival of neutrophils to sites of infection may also influence the outcome of disease progression by regulating other types of immune cells. Bi Y *et al*. recently reported that production of IL-17A by neutrophils coordinates the antimicrobial activity of neutrophils and macrophages against *Y*. *pestis* infection during pneumonic plague [66]. In addition, the secretion of neutrophil-derived granule proteins and antibacterial peptides was associated with the migration of inflammatory monocytes to the site of infection [67, 68]. The therapeutic potential of the early recruitment of neutrophils to the lungs may also be attributed to the involvement of these cells in disease resolution and anti-inflammatory processes. Neutrophils have recently been shown to play an important role in the repair of damaged tissue through the expression of MMP9, which degrades intracellular matrix (ICM) components, thus promoting the removal of damage-associated molecular pattern (DAMP)-containing ICM proteins released from damaged cells (Reviewed in [69]).

Taken together, this study highlights (a) the complex virulence mechanisms employed by *Y*. *pestis* to minimize its early encounter with neutrophils in the lungs following airway infection and (b) the beneficial effect of modulating neutrophil chemotaxis into the lungs at the early stage of *Y*. *pestis* infection by treatment with chemoattractants. This therapeutic approach could be useful for improving treatments against plague as well as other pathogens that suppress the recruitment of neutrophils to sites of infection. The existence of antibiotic-resistant *Y*. *pestis* strains [70] further emphasizes the importance of modulating the host defense for the treatment of plague infections.

## Materials and Methods

### Ethics statement

This study was carried out in strict accordance with the recommendations for the Care and Use of Laboratory Animals of the National Institute of Health. All animal experiments were performed in accordance with Israeli law and were approved by the Ethics Committee for animal experiments at the Israel Institute for Biological Research (Permit Numbers: IACUC-IIBR M-07-2012, IACUC-IIBR M-28-2013). During the experiments, the mice were monitored daily. Humane endpoints were used in our survival studies. Mice exhibiting loss of the righting reflex were euthanized by cervical dislocation. Analgesics were not used, as they may have affected the experimental outcomes of the studies.

### Bacterial strains and culture conditions

The following *Y*. *pestis* strains were used in this study: the *Y*. *pestis* virulent strain Kimberley53 (Kim53) [71], the avirulent Kim53Δ70Δ10 strain that is spontaneously cured for pPCP1 and pCD1 [72], Kim53 deleted for YopJ (Kim53ΔYopJ) [73] and the Kim53 deleted for YopH (Kim53ΔYopH) (Constructed by Zauberman A, replacing the *yopH* gene with a kanamycin resistance cassette as described in [73]). The *Y*. *pestis* vaccine strain EV76 [71], EV76 spontaneously cured for pCD1 (EV76Δp70) [74], EV76ΔYopJ [46], and the EV76 deleted mutants EV76ΔYopK, EV76ΔYopE, EV76ΔYopH (Constructed by Zauberman A., replacing the *yopK*, *yopE* and *yopH* genes with a kanamycin resistance cassette as described in [73]). The strains were routinely grown on brain heart infusion agar (BHIA, BD, MD USA) for 48 hr at 28°C. The *Y*. *pestis* Yop-deleted strains were grown on BHIA supplemented with 100 μg/ml kanamycin (Sigma-Aldrich, Israel).

### Construction of *Y*. *pestis* Yop mutants

Deletion mutagenesis of the *Y*. *pestis* Kim53 and EV76 strains was performed by replacing the central region of the genes with a kanamycin resistance cassette (Pharmacia) by homologous recombination. The protocol used was based on previously established methodologies [75, 76]. The linear PCR fragment in which kanamycin sequences were flanked by *yop* sequences was electroporated into *Y*. *pestis* bacteria expressing the λ phage *red* system from pKOBEG::*sac*B (generous gift from Dr. E. Carniel). Electroporation was performed in 10% glycerol and 10% PEG-8000 (Sigma-Aldrich, Israel), and the bacteria were incubated in HIB for 2 h at 28°C. Transformants were selected on BHIA containing 50 μg/ml kanamycin, and then the pKOBEG::*sac*B plasmid was removed from the bacteria by growing the bacteria on BHIA supplemented with 10% sucrose. The expected knockout phenotype was verified by PCR and Western blot analyses.

### Animal infections

Female C57BL/6 mice (6–10 weeks old) were purchased from Harlan Laboratories (Rehovot, Israel) and maintained under defined flora conditions at the animal facilities of the Israel Institute for Biological Research. The i.n. infections were performed as described previously [77]. Briefly, bacterial colonies were harvested and diluted in heart infusion broth (HIB) (BD, USA) supplemented with 0.2% xylose and 2.5 mM CaCl_2_ (Sigma-Aldrich, Israel) to an OD_660_ of 0.01 and grown for 22 h at 28°C in a shaker (100 rpm). At the end of the incubation period, the cultures were washed, diluted in PBS solution to the required infectious dose and quantified by counting colony forming units (cfu) after plating and incubating on BHIA plates (48 hr at 28°C). Prior to infection, mice were anesthetized with a mixture of 0.5% ketamine HCl and 0.1% xylazine and then infected i.n. with 35 μl/mouse of the bacterial suspension, whereas naïve mice were instilled i.n. with PBS only. The intranasal LD_50_ of the Kim53 strain under these conditions is 1,100 cfu. LD_50_ values were calculated according to the method described by Reed and Muench [78].

### Combined treatment with G-CSF, KC and MIP-2 and depletion of neutrophils

Three days prior to infection, mice received daily subcutaneous injections of recombinant G-CSF (rhG-CSF 300 μg/kg/Nupogen 48 MU/0.5 ml, Roche Applied Science) for 5 consecutive days. Six hours after i.n. infection with *Y*. *pestis* Kim53, mice were euthanized, and 1 μg of each recombinant KC and MIP-2 (recombinant MCXCL1/KC, recombinant MCXCL2/MIP-2, R&D Systems), diluted in 25 μl of PBS, or 25 μl of PBS alone (sham) were instilled i.n. Mice were either sacrificed and analyzed 24 hpi or followed to observe the rates of morbidity and mortality. For the depletion of neutrophils, 100 μg purified anti-Ly-6G antibody clone 1A8 (Biolegend, USA) diluted in 300 μl PBS was administered intraperitoneally twice at 24 hr prior to infection and 3 hpi.

### Cell infection

The murine alveolar macrophage cell line MH-S was obtained from ATCC. TC-1 is a tumor cell line derived from primary lung epithelial cells of C57BL/6 mice[79]. This cell line was a kind gift from the laboratory of Prof. T.C. Wu (Johns Hopkins University). Both cell-lines were grown in RPMI 1640 medium supplemented with 10 mM HEPES, 2 mM L-glutamine, 1 mM sodium pyruvate, 0.1 mM non-essential amino-acids and 10% fetal bovine serum. Cell cultures were maintained at 37°C with 5% CO_2_. Cell infection studies were performed as previously described [73]. Briefly, bacteria were grown by shaking (150 rpm) for 22 h at 28°C in HIB. The resulting cultures were diluted in HIB medium to OD_660_ 0.05 and allowed to grow for 3 h at 37°C (100 rpm). Bacteria were harvested, washed once and re-suspended in complete RPMI supplemented with 10% fetal calf serum and added to the cells at a multiplicity of infection (MOI) of 50. Bacteria were adhered onto the cells by centrifugation at 130 g for 5 min followed by incubation for an additional 1 h at 37°C and 5% CO_2_. Gentamicin was then added to the cultures to a final concentration of 50 mg/ml, and the cultures were incubated for an additional 4 h before using the cells for RNA extraction and RT-PCR analysis and the media for ELISA.

### Flow cytometry analysis

To prepare lung cell suspensions, mice were euthanized, and blood was withdrawn from the heart using a heparinized syringe. Lungs were then removed and placed on a 70-μm nylon cell strainer (BD Falcon,USA) dipped in 2 ml PBS containing 1% protease inhibitor cocktail (Sigma-Aldrich, Israel). Cell suspensions were pelleted at 260 g for 10 min at 4°C, fixed in 4% paraformaldehyde in PBS for 1 h at room temperature and washed twice in flow cytometry buffer. Neutrophils (CD11b^+^/Gr-1^high^) were stained with PerCP-Cy5.5-conjugated anti-mouse CD11b antibody (clone M1/70) (eBioscience, USA) and APC-conjugated anti-mouse Ly-6G (Gr-1) antibody (clone RB6-8C5) (eBioscience, USA). CXCR2 staining was performed with PE-conjugated anti-CXCR2 antibodies (clone 242216) (R&D, USA). Cells were stained using standard protocols with appropriate matched isotype control antibodies. The analysis was performed using a FACSCalibur flow cytometer with CellQuest Pro software (BD Bioscience, USA).

### RT-PCR and quantitative PCR analysis

Lung cell suspensions were prepared as previously described. Total RNA was extracted using Tri-reagent (Sigma-Aldrich, Israel) according to the manufacturer’s instructions. Two micrograms of total RNA were reverse-transcribed using Moloney murine leukemia virus reverse transcriptase and oligo-dT primers (Promega, USA). Quantitative PCR analysis was performed using an ABI 7500 instrument (Applied Biosystems, USA) with SYBR green PCR master mix (Applied Biosystems, USA). The fold change in the quantity of gene transcripts was measured and compared to the hypoxanthine phosphoribosyl transferase (HPRT) gene using the comparative (-2^ΔΔCt^) method. Forty cycles of PCR amplification were performed in duplicate for each primer set. Primer sequences used are listed in Table 1.

**Table 1:** Sequences of the primers used in this study.

Mouse gene	Forward 5’-3’	Reverse 5’-3’
**KC** NM_008176	CAATGAGCTGCGCTGTCAGT	CAAGGGAGCTTCAGGGTCAA
**MIP-2** NM_009140	CCTGCCGGCTCCTCAGT	CTTTTTGACCGCCCTTGAGA
**G-CSF** NM_009971	CCTGGAGCAAGTGAGGAAGATC	AGAGAGTGGCCCAGCAACAC
**MMP8** NM_008611	CACACACAGCTTGCCAATGC	TCCCAGTCTCTGCTAAGCTGAAG
**ICAM-1** NM_010493	TCCGGACTTTCGATCTTCCA	GAGCTTCAGAGGCAGGAAACA
**VCAM-1** NM_011693	CTTGGGAGCCTCAACGGTACT	GCCCGTAGTGCTGCAAGTG
**P-selectin** NM_011347	CAACGAGCCCAACAACAAGA	CGATGCACTCCCCTTGGTT
**E-selectin** NM_011345	CTCCAGGTGAACCAAACAACA	TGACAACTGCAGGATGCATT
**HPRT** NM_013556	AGTACAGCCCCAAAATGG	TCCTTTTCACCAGCAAGCT

### Chemokines and total MMP9 analysis

Blood was collected and centrifuged at 260 g for 10 min, and the plasma was collected, filtered and stored at -70°C. Bronchoalveolar lavage fluid (BALF) was collected by exposing the trachea and injecting, and then removing twice, a total of 1 ml PBS containing 1% protease inhibitor cocktail (Sigma-Aldrich, Israel). BALF was then filtered and stored at -70°C. Before analysis, samples were centrifuged again at 13,000 g for 5 min. The levels of KC, MIP-2 and G-CSF in the plasma and BALF and the levels of MMP9 in the BALF, were measured by enzyme-linked immunosorbent assay (ELISA) according to the manufacturer’s protocol (R&D Systems, MN, USA).

### Gelatin zymography for MMP9 activity

Gelatin zymography for MMP9 activity was performed as previously described [80]. Briefly, whole lung supernatants were mixed with a non-reducing sample buffer, and an equal amount was loaded onto 10% SDS-polyacrylamide gels co-polymerized with 1 mg/ml gelatin derived from porcine skin (Sigma-aldrich, Israel). After electrophoresis, the gels were washed for 30 min in Triton X-100, followed by 3 washes with H_2_O and incubation at 37°C for 16 hr in developing buffer. The gels were then stained with SeeBand Forte (Gene Bio-Application Ltd) until clear bands appeared, indicating the presence of MMP9 activity. Conditioned media of HT-1080 cells secreting MMP9 served as a control.

### Transwell-migration assay

Transwell-migration assays were performed as previously described [81]. Briefly, total BM cells were extracted from the femur and tibias of naïve mice and suspended in complete RPMI media. Prior to the migration assay, the total BM cells were labeled with the neutrophil markers Gr-1 and CD11b to evaluate the levels of neutrophils. The cells were counted, and 250,000 cells/100 μl were allowed to migrate towards a total of 600 μl media containing 150 μl of lung supernatant through 24-well filters with a pore size of 5 μm (Corning, NY, USA) at 37°C for 3 hr. The cells were then collected and counted using flow cytometry. In parallel, a portion of the migrated cells was labeled with Gr-1 and CD11b to evaluate the number of migrated cells. The percentage of migrated cells was then calculated by comparing the number of neutrophils before and after migration.

### Immunoblot for MMP8

Bronchoalveolar lavage fluid (BALF) was collected as previously described, and equal amount of BALF samples were subjected to 10% SDS-PAGE followed by immunoblot with anti MMP8 polyclonal antibody (Proteintech cat:17874-1-AP).

### Statistical analysis

Statistical significance was determined using the nonparametric Mann-Whitney test. A Kaplan-Meier survival estimate of treated and non-treated mice (of at least 10 mice per group) was also performed. Calculations were made using GraphPad Prism software.

## Supporting Information

S1 Fig Survival curves. C57BL/6J mice were infected i.n. with 1x10^5^ cfu of the fully virulent *Y*. *pestis* strain Kim53 (black line) or with 1x10^5^ cfu of the Kim53ΔYopJ mutant (red line). C57BL/6J mice were treated for 5 consecutive days with a daily subcutaneous injection of G-CSF. At day 3 of the G-CSF treatment, recombinant KC and MIP-2 proteins (1μg/mouse, each) were i.n. administered to the G-CSF-treated mice (blue line). n = 5 for each group of mice.
(TIF)

S2 Fig *In vitro* infection of alveolar macrophages and lung epithelial cells with EV76 *Y*. *pestis* strains. *In-vitro* infection of alveolar macrophages (MH-S) (A) and lung-derived epithelial cell lines (TC-1) (B) with 50 MOI of the attenuated *Y*. *pestis* strain EV76 and its Yop-depleted derivatives: EV76ΔYopK (ΔK), EV76ΔYopE (ΔE), EV76ΔYopJ (ΔJ), EV76ΔYopH (ΔH) and EV76ΔpCD1 (Δ70). The mRNA levels of the chemokines KC (A), MIP-2 (A and B) and G-CSF (B) were quantified using qPCR analysis and are presented as the fold change relative to the wild-type *Y*. *pestis* EV76.
(TIF)

S3 Fig CXCR2 expression on blood circulation neutrophils. Expression of cell surface CXCR2 on circulation neutrophils isolated from the peripheral blood of C57BL/6 mice at 24 hpi with 1×10^5^ cfu of the virulent *Y*. *pestis* strain Kim53 in comparison to naive mice. Representative FACS histogram analysis showing CXCR2 expression on Gr-1high/CD11b+ peripheral blood neutrophils at 24 hpi (purple area), compared to naïve mice (green line). The average CXCR2 Geo-mean levels are indicated.
(TIF)

S4 Fig Expression of E/P-selectins in the lungs of GKM-treated mice infected i.n. with *Y*. *pestis*. C57BL/6 mice were infected i.n. with 1×10^5^ cfu (100 LD_50_) of the virulent *Y*. *pestis* strain Kim53. The mRNA of sham and GKM—treated mice was purified from the infected lungs at 24 hpi and subjected to qPCR analysis of E/P-selectin gene expression. The results are presented as the means ± SEM (*p<0.05). mRNA levels are presented as fold change relative to sham-treated mice.
(TIF)

## References

[1] Craig A, Mai J, Cai S, Jeyaseelan S. Neutrophil recruitment to the lungs during bacterial pneumonia. Infect Immun. 2009;77(2):568–75. Epub 2008/11/19. IAI.00832-08 [pii]. 10.1128/IAI.00832-08 19015252 PMC2632043

[2] King KY, Goodell MA. Inflammatory modulation of HSCs: viewing the HSC as a foundation for the immune response. Nat Rev Immunol. 2011;11(10):685–92. Epub 2011/09/10. 10.1038/nri3062 21904387 PMC4154310

[3] Rogers HW, Unanue ER. Neutrophils are involved in acute, nonspecific resistance to Listeria monocytogenes in mice. Infect Immun. 1993;61(12):5090–6. 8225586 10.1128/iai.61.12.5090-5096.1993 PMC281287

[4] Borregaard N. Neutrophils, from marrow to microbes. Immunity. 2010;33(5):657–70. 10.1016/j.immuni.2010.11.011 21094463

[5] Bozic CR, Kolakowski LF Jr., Gerard NP, Garcia-Rodriguez C, von Uexkull-Guldenband C, Conklyn MJ, et al Expression and biologic characterization of the murine chemokine KC. J Immunol. 1995;154(11):6048–57. Epub 1995/06/01. 7751647

[6] Strieter RM, Kunkel SL. Acute lung injury: the role of cytokines in the elicitation of neutrophils. J Investig Med. 1994;42(4):640–51. Epub 1994/12/01. 8521027

[7] Williams MR, Azcutia V, Newton G, Alcaide P, Luscinskas FW. Emerging mechanisms of neutrophil recruitment across endothelium. Trends Immunol. 2011;32(10):461–9. Epub 2011/08/16. 10.1016/j.it.2011.06.009 21839681 PMC3185121

[8] Mocsai A. Diverse novel functions of neutrophils in immunity, inflammation, and beyond. J Exp Med. 2013;210(7):1283–99. Epub 2013/07/05. 10.1084/jem.20122220 23825232 PMC3698517

[9] Mizgerd JP. Acute Lower Respiratory Tract Infection. New England Journal of Medicine. 2008;358(7):716–27. 10.1056/NEJMra074111 18272895 PMC2711392

[10] Nathan C. Neutrophils and immunity: challenges and opportunities. Nat Rev Immunol. 2006;6(3):173–82. Epub 2006/02/25. 16498448 10.1038/nri1785

[11] Tsai WC, Strieter RM, Mehrad B, Newstead MW, Zeng X, Standiford TJ. CXC chemokine receptor CXCR2 is essential for protective innate host response in murine Pseudomonas aeruginosa pneumonia. Infect Immun. 2000;68(7):4289–96. 10858247 10.1128/iai.68.7.4289-4296.2000 PMC101748

[12] Tateda K, Moore TA, Newstead MW, Tsai WC, Zeng X, Deng JC, et al Chemokine-dependent neutrophil recruitment in a murine model of Legionella pneumonia: potential role of neutrophils as immunoregulatory cells. Infect Immun. 2001;69(4):2017–24. Epub 2001/03/20. 11254553 10.1128/IAI.69.4.2017-2024.2001 PMC98125

[13] Matsuzaki G, Umemura M. Interleukin-17 as an effector molecule of innate and acquired immunity against infections. Microbiol Immunol. 2007;51(12):1139–47. 18094532 10.1111/j.1348-0421.2007.tb04008.x

[14] Eisele NA, Lee-Lewis H, Besch-Williford C, Brown CR, Anderson DM. Chemokine receptor CXCR2 mediates bacterial clearance rather than neutrophil recruitment in a murine model of pneumonic plague. Am J Pathol. 2011;178(3):1190–200. 10.1016/j.ajpath.2010.11.067 21356370 PMC3070576

[15] Laws TR, Davey MS, Titball RW, Lukaszewski R. Neutrophils are important in early control of lung infection by Yersinia pestis. Microbes Infect. 2010;12(4):331–5. 10.1016/j.micinf.2010.01.007 20114086

[16] Perry RD, Fetherston JD. Yersinia pestis—etiologic agent of plague. Clin Microbiol Rev. 1997;10(1):35–66. 8993858 10.1128/cmr.10.1.35 PMC172914

[17] Kool JL. Risk of person-to-person transmission of pneumonic plague. Clin Infect Dis. 2005;40(8):1166–72. 15791518 10.1086/428617

[18] Pollitzer R. Plague studies. IX. Epidemiology. Bull World Health Organ. 1954;9(1):131–70. PMC2542107 13082391

[19] Inglesby TV, Dennis DT, Henderson DA, Bartlett JG, Ascher MS, Eitzen E, et al Plague as a biological weapon: medical and public health management. Working Group on Civilian Biodefense. Jama. 2000;283(17):2281–90. 10807389 10.1001/jama.283.17.2281

[20] Agar SL, Sha J, Foltz SM, Erova TE, Walberg KG, Parham TE, et al Characterization of a mouse model of plague after aerosolization of Yersinia pestis CO92. Microbiology. 2008;154(Pt 7):1939–48. 10.1099/mic.0.2008/017335-0 18599822

[21] Bubeck SS, Cantwell AM, Dube PH. Delayed inflammatory response to primary pneumonic plague occurs in both outbred and inbred mice. Infect Immun. 2007;75(2):697–705. 17101642 10.1128/IAI.00403-06 PMC1828510

[22] Lathem WW, Crosby SD, Miller VL, Goldman WE. Progression of primary pneumonic plague: a mouse model of infection, pathology, and bacterial transcriptional activity. Proc Natl Acad Sci U S A. 2005;102(49):17786–91. 16306265 10.1073/pnas.0506840102 PMC1308902

[23] Price PA, Jin J, Goldman WE. Pulmonary infection by Yersinia pestis rapidly establishes a permissive environment for microbial proliferation. Proc Natl Acad Sci U S A. 2012;109(8):3083–8. 10.1073/pnas.1112729109 22308352 PMC3286930

[24] Cornelis GR, Wolf-Watz H. The Yersinia Yop virulon: a bacterial system for subverting eukaryotic cells. Mol Microbiol. 1997;23(5):861–7. Epub 1997/03/01. 9076724 10.1046/j.1365-2958.1997.2731623.x

[25] Mota LJ, Cornelis GR. The bacterial injection kit: type III secretion systems. Ann Med. 2005;37(4):234–49. Epub 2005/07/16. 16019722 10.1080/07853890510037329

[26] Viboud GI, Bliska JB. Yersinia outer proteins: role in modulation of host cell signaling responses and pathogenesis. Annu Rev Microbiol. 2005;59:69–89. Epub 2005/04/26. 15847602 10.1146/annurev.micro.59.030804.121320

[27] Shannon JG, Hasenkrug AM, Dorward DW, Nair V, Carmody AB, Hinnebusch BJ. Yersinia pestis subverts the dermal neutrophil response in a mouse model of bubonic plague. MBio. 2013;4(5):e00170–13. Epub 2013/08/29. 10.1128/mBio.00170-13 23982068 PMC3760243

[28] Welkos S, Friedlander A, McDowell D, Weeks J, Tobery S. V antigen of Yersinia pestis inhibits neutrophil chemotaxis. Microb Pathog. 1998;24(3):185–96. 9514641 10.1006/mpat.1997.0188

[29] Pechous RD, Sivaraman V, Price PA, Stasulli NM, Goldman WE. Early host cell targets of Yersinia pestis during primary pneumonic plague. PLoS Pathog. 2013;9(10):e1003679 Epub 2013/10/08. 10.1371/journal.ppat.1003679 24098126 PMC3789773

[30] Vagima Y, Levy Y, Gur D, Tidhar A, Aftalion M, Abramovich H, et al Early sensing of Yersinia pestis airway infection by bone marrow cells. Front Cell Infect Microbiol. 2012;2:143 Epub 2012/11/29. 10.3389/fcimb.2012.00143 23189271 PMC3505838

[31] Greenlee KJ, Werb Z, Kheradmand F. Matrix metalloproteinases in lung: multiple, multifarious, and multifaceted. Physiol Rev. 2007;87(1):69–98. Epub 2007/01/24. 17237343 10.1152/physrev.00022.2006 PMC2656382

[32] Furze RC, Rankin SM. Neutrophil mobilization and clearance in the bone marrow. Immunology. 2008;125(3):281–8. Epub 2009/01/09. IMM2950 [pii]. 10.1111/j.1365-2567.2008.02950.x 19128361 PMC2669132

[33] Bosio CM, Goodyear AW, Dow SW. Early interaction of Yersinia pestis with APCs in the lung. J Immunol. 2005;175(10):6750–6. Epub 2005/11/08. 16272331 10.4049/jimmunol.175.10.6750

[34] Aird WC. Phenotypic heterogeneity of the endothelium: I. Structure, function, and mechanisms. Circ Res. 2007;100(2):158–73. Epub 2007/02/03. 17272818 10.1161/01.RES.0000255691.76142.4a

[35] Bliska JB, Wang X, Viboud GI, Brodsky IE. Modulation of innate immune responses by Yersinia type III secretion system translocators and effectors. Cell Microbiol. 2013;15(10):1622–31. Epub 2013/07/10. 10.1111/cmi.12164 23834311 PMC3788085

[36] Navarro L, Alto NM, Dixon JE. Functions of the Yersinia effector proteins in inhibiting host immune responses. Curr Opin Microbiol. 2005;8(1):21–7. Epub 2005/02/08. 15694853 10.1016/j.mib.2004.12.014

[37] Trosky JE, Liverman AD, Orth K. Yersinia outer proteins: Yops. Cell Microbiol. 2008;10(3):557–65. Epub 2007/12/18. 10.1111/j.1462-5822.2007.01109.x . 18081726

[38] Phillipson M, Kubes P. The neutrophil in vascular inflammation. Nat Med. 2011;17(11):1381–90. Epub 2011/11/09. 10.1038/nm.2514 22064428 PMC7095830

[39] Scott DW, Patel RP. Endothelial heterogeneity and adhesion molecules N-glycosylation: implications in leukocyte trafficking in inflammation. Glycobiology. 2013;23(6):622–33. Epub 2013/03/01. 10.1093/glycob/cwt014 23445551

[40] Denecker G, Totemeyer S, Mota LJ, Troisfontaines P, Lambermont I, Youta C, et al Effect of low- and high-virulence Yersinia enterocolitica strains on the inflammatory response of human umbilical vein endothelial cells. Infect Immun. 2002;70(7):3510–20. Epub 2002/06/18. 12065490 10.1128/IAI.70.7.3510-3520.2002 PMC128109

[41] Cornelis GR. Yersinia type III secretion: send in the effectors. J Cell Biol. 2002;158(3):401–8. 12163464 10.1083/jcb.200205077 PMC2173816

[42] Viboud GI, Mejia E, Bliska JB. Comparison of YopE and YopT activities in counteracting host signalling responses to Yersinia pseudotuberculosis infection. Cell Microbiol. 2006;8(9):1504–15. Epub 2006/08/23. 16922868 10.1111/j.1462-5822.2006.00729.x

[43] Viboud GI, So SS, Ryndak MB, Bliska JB. Proinflammatory signalling stimulated by the type III translocation factor YopB is counteracted by multiple effectors in epithelial cells infected with Yersinia pseudotuberculosis. Mol Microbiol. 2003;47(5):1305–15. 12603736 10.1046/j.1365-2958.2003.03350.x

[44] Palmer LE, Hobbie S, Galan JE, Bliska JB. YopJ of Yersinia pseudotuberculosis is required for the inhibition of macrophage TNF-alpha production and downregulation of the MAP kinases p38 and JNK. Mol Microbiol. 1998;27(5):953–65. Epub 1998/04/16. 9535085 10.1046/j.1365-2958.1998.00740.x

[45] Ruckdeschel K. Immunomodulation of macrophages by pathogenic Yersinia species. Arch Immunol Ther Exp (Warsz). 2002;50(2):131–7. Epub 2002/05/23. 12022702

[46] Zauberman A, Cohen S, Mamroud E, Flashner Y, Tidhar A, Ber R, et al Interaction of Yersinia pestis with macrophages: limitations in YopJ-dependent apoptosis. Infect Immun. 2006;74(6):3239–50. Epub 2006/05/23. 16714551 10.1128/IAI.00097-06 PMC1479247

[47] Zhou L, Tan A, Hershenson MB. Yersinia YopJ inhibits pro-inflammatory molecule expression in human bronchial epithelial cells. Respir Physiol Neurobiol. 2004;140(1):89–97. Epub 2004/04/28. 15109931 10.1016/j.resp.2003.12.003

[48] Sauvonnet N, Lambermont I, van der Bruggen P, Cornelis GR. YopH prevents monocyte chemoattractant protein 1 expression in macrophages and T-cell proliferation through inactivation of the phosphatidylinositol 3-kinase pathway. Mol Microbiol. 2002;45(3):805–15. Epub 2002/07/26. 12139625 10.1046/j.1365-2958.2002.03053.x

[49] Cantwell AM, Bubeck SS, Dube PH. YopH inhibits early pro-inflammatory cytokine responses during plague pneumonia. BMC Immunol. 2010;11:29 Epub 2010/06/23. 10.1186/1471-2172-11-29 20565713 PMC2894752

[50] Orth K, Xu Z, Mudgett MB, Bao ZQ, Palmer LE, Bliska JB, et al Disruption of signaling by Yersinia effector YopJ, a ubiquitin-like protein protease. Science. 2000;290(5496):1594–7. Epub 2000/11/25. 11090361 10.1126/science.290.5496.1594

[51] Zhou H, Monack DM, Kayagaki N, Wertz I, Yin J, Wolf B, et al Yersinia virulence factor YopJ acts as a deubiquitinase to inhibit NF-kappa B activation. J Exp Med. 2005;202(10):1327–32. Epub 2005/11/23. 16301742 10.1084/jem.20051194 PMC2212976

[52] Mittal R, Peak-Chew SY, McMahon HT. Acetylation of MEK2 and I kappa B kinase (IKK) activation loop residues by YopJ inhibits signaling. Proc Natl Acad Sci U S A. 2006;103(49):18574–9. Epub 2006/11/23. 17116858 10.1073/pnas.0608995103 PMC1654131

[53] Mukherjee S, Keitany G, Li Y, Wang Y, Ball HL, Goldsmith EJ, et al Yersinia YopJ acetylates and inhibits kinase activation by blocking phosphorylation. Science. 2006;312(5777):1211–4. Epub 2006/05/27. 16728640 10.1126/science.1126867

[54] Pahl HL. Activators and target genes of Rel/NF-kappaB transcription factors. Oncogene. 1999;18(49):6853–66. Epub 1999/12/22. 10602461 10.1038/sj.onc.1203239

[55] Tebo JM, Datta S, Kishore R, Kolosov M, Major JA, Ohmori Y, et al Interleukin-1-mediated stabilization of mouse KC mRNA depends on sequences in both 5'- and 3'-untranslated regions. J Biol Chem. 2000;275(17):12987–93. Epub 2000/04/25. 10777600 10.1074/jbc.275.17.12987

[56] Mizgerd JP, Lupa MM, Spieker MS. NF-kappaB p50 facilitates neutrophil accumulation during LPS-induced pulmonary inflammation. BMC Immunol. 2004;5:10 Epub 2004/06/11. 15189567 10.1186/1471-2172-5-10 PMC449706

[57] Lemaitre N, Sebbane F, Long D, Hinnebusch BJ. Yersinia pestis YopJ suppresses tumor necrosis factor alpha induction and contributes to apoptosis of immune cells in the lymph node but is not required for virulence in a rat model of bubonic plague. Infect Immun. 2006;74(9):5126–31. Epub 2006/08/24. 16926404 10.1128/IAI.00219-06 PMC1594864

[58] Straley SC, Bowmer WS. Virulence genes regulated at the transcriptional level by Ca2+ in Yersinia pestis include structural genes for outer membrane proteins. Infect Immun. 1986;51(2):445–54. Epub 1986/02/01. 3002984 10.1128/iai.51.2.445-454.1986 PMC262351

[59] Zauberman A, Velan B, Mamroud E, Flashner Y, Shafferman A, Cohen S. Disparity between Yersinia pestis and Yersinia enterocolitica O:8 in YopJ/YopP-dependent functions. Adv Exp Med Biol. 2007;603:312–20. Epub 2007/10/31. 17966427 10.1007/978-0-387-72124-8_28

[60] Lathem WW, Price PA, Miller VL, Goldman WE. A plasminogen-activating protease specifically controls the development of primary pneumonic plague. Science. 2007;315(5811):509–13. Epub 2007/01/27. 17255510 10.1126/science.1137195

[61] Caulfield AJ, Walker ME, Gielda LM, Lathem WW. The Pla protease of Yersinia pestis degrades fas ligand to manipulate host cell death and inflammation. Cell Host Microbe. 2014;15(4):424–34. Epub 2014/04/12. 10.1016/j.chom.2014.03.005 24721571 PMC4020149

[62] Gorgen I, Hartung T, Leist M, Niehorster M, Tiegs G, Uhlig S, et al Granulocyte colony-stimulating factor treatment protects rodents against lipopolysaccharide-induced toxicity via suppression of systemic tumor necrosis factor-alpha. J Immunol. 1992;149(3):918–24. Epub 1992/08/01. 1378868

[63] Roberts AW. G-CSF: a key regulator of neutrophil production, but that's not all! Growth Factors. 2005;23(1):33–41. Epub 2005/07/16. 16019425 10.1080/08977190500055836

[64] Andreasen C, Carbonetti NH. Pertussis toxin inhibits early chemokine production to delay neutrophil recruitment in response to Bordetella pertussis respiratory tract infection in mice. Infect Immun. 2008;76(11):5139–48. Epub 2008/09/04. 10.1128/IAI.00895-08 18765723 PMC2573337

[65] Sun K, Salmon SL, Lotz SA, Metzger DW. Interleukin-12 promotes gamma interferon-dependent neutrophil recruitment in the lung and improves protection against respiratory Streptococcus pneumoniae infection. Infect Immun. 2007;75(3):1196–202. Epub 2007/01/11. 17210665 10.1128/IAI.01403-06 PMC1828591

[66] Bi Y, Zhou J, Yang H, Wang X, Zhang X, Wang Q, et al IL-17A produced by neutrophils protects against pneumonic plague through orchestrating IFN-gamma-activated macrophage programming. J Immunol. 2014;192(2):704–13. Epub 2013/12/18. 10.4049/jimmunol.1301687 24337746

[67] Soehnlein O. Direct and alternative antimicrobial mechanisms of neutrophil-derived granule proteins. J Mol Med (Berl). 2009;87(12):1157–64. Epub 2009/07/31. 10.1007/s00109-009-0508-6 19641860

[68] Soehnlein O, Zernecke A, Weber C. Neutrophils launch monocyte extravasation by release of granule proteins. Thromb Haemost. 2009;102(2):198–205. Epub 2009/08/05. 10.1160/TH08-11-0720 19652869

[69] Kolaczkowska E, Kubes P. Neutrophil recruitment and function in health and inflammation. Nat Rev Immunol. 2013;13(3):159–75. Epub 2013/02/26. 10.1038/nri3399 23435331

[70] Galimand M, Carniel E, Courvalin P. Resistance of Yersinia pestis to antimicrobial agents. Antimicrob Agents Chemother. 2006;50(10):3233–6. Epub 2006/09/29. 17005799 10.1128/AAC.00306-06 PMC1610074

[71] Ben-Gurion R, Shafferman A. Essential virulence determinants of different Yersinia species are carried on a common plasmid. Plasmid. 1981;5(2):183–7. Epub 1981/03/01. 7243971 10.1016/0147-619x(81)90019-6

[72] Flashner Y, Mamroud E, Tidhar A, Ber R, Aftalion M, Gur D, et al Generation of Yersinia pestis attenuated strains by signature-tagged mutagenesis in search of novel vaccine candidates. Infect Immun. 2004;72(2):908–15. Epub 2004/01/27. 14742535 10.1128/IAI.72.2.908-915.2004 PMC321629

[73] Zauberman A, Tidhar A, Levy Y, Bar-Haim E, Halperin G, Flashner Y, et al Yersinia pestis endowed with increased cytotoxicity is avirulent in a bubonic plague model and induces rapid protection against pneumonic plague. PLoS One. 2009;4(6):e5938 10.1371/journal.pone.0005938 19529770 PMC2691952

[74] Flashner Y, Mamroud E, Tidhar A, Ber R, Aftalion M, Gur D, et al Identification of genes involved in Yersinia pestis virulence by signature-tagged mutagenesis. Adv Exp Med Biol. 2003;529:31–3. Epub 2003/05/22. 12756723 10.1007/0-306-48416-1_5

[75] Datsenko KA, Wanner BL. One-step inactivation of chromosomal genes in Escherichia coli K-12 using PCR products. Proc Natl Acad Sci U S A. 2000;97(12):6640–5. Epub 2000/06/01. 10829079 10.1073/pnas.120163297 PMC18686

[76] Derbise A, Lesic B, Dacheux D, Ghigo JM, Carniel E. A rapid and simple method for inactivating chromosomal genes in Yersinia. FEMS Immunol Med Microbiol. 2003;38(2):113–6. Epub 2003/09/18. 13129645 10.1016/S0928-8244(03)00181-0

[77] Tidhar A, Flashner Y, Cohen S, Levi Y, Zauberman A, Gur D, et al The NlpD lipoprotein is a novel Yersinia pestis virulence factor essential for the development of plague. PLoS One. 2009;4(9):e7023 10.1371/journal.pone.0007023 19759820 PMC2736372

[78] Reed LJ MH. A simple method of estimating fifty percent endpoints. TheAmerican Journal of Hygiene. 1938;27:493–7.

[79] Lin KY, Guarnieri FG, Staveley-O'Carroll KF, Levitsky HI, August JT, Pardoll DM, et al Treatment of established tumors with a novel vaccine that enhances major histocompatibility class II presentation of tumor antigen. Cancer Res. 1996;56(1):21–6. Epub 1996/01/01. 8548765

[80] Vagima Y, Avigdor A, Goichberg P, Shivtiel S, Tesio M, Kalinkovich A, et al MT1-MMP and RECK are involved in human CD34+ progenitor cell retention, egress, and mobilization. J Clin Invest. 2009;119(3):492–503. 10.1172/JCI36541 19197139 PMC2648678

[81] Vagima Y, Lapid K, Kollet O, Goichberg P, Alon R, Lapidot T. Pathways implicated in stem cell migration: the SDF-1/CXCR4 axis. Methods Mol Biol. 2011;750:277–89. Epub 2011/05/28. 10.1007/978-1-61779-145-1_19 21618098

