# Supplementary figures and images for: Circumventing Y. pestis Virulence by Early Recruitment of Neutrophils to the Lungs during Pneumonic Plague
Source: PLoS Pathog. 2015 May 14;11(5):e1004893. doi: 10.1371/journal.ppat.1004893 (PMC4431741; doi:10.1371/journal.ppat.1004893)

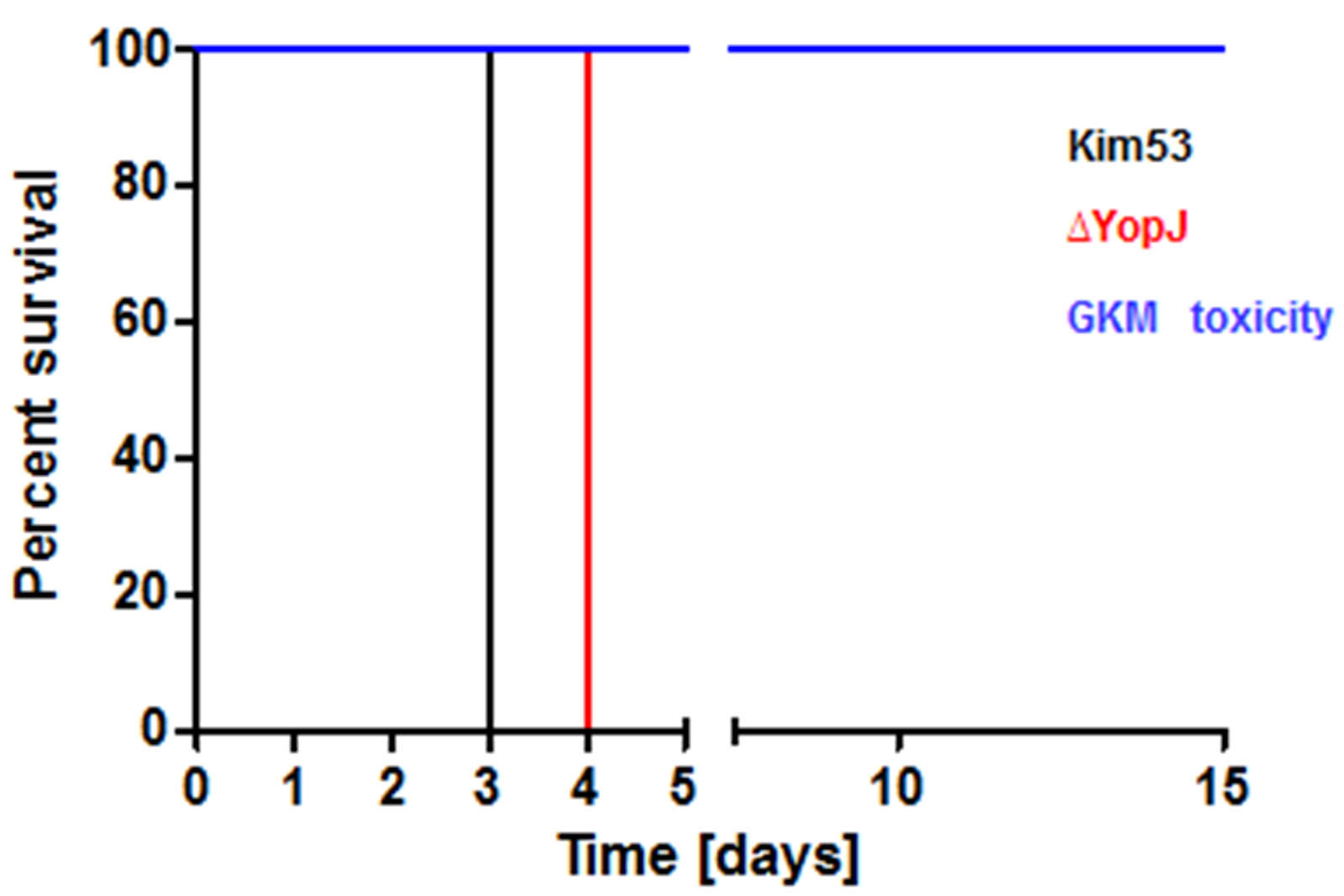

Supplement: S1 Fig — C57BL/6J mice were infected i.n. with 1x10^5^ cfu of the fully virulent Y. pestis strain Kim53 (black line) or with 1x10^5^ cfu of the Kim53ΔYopJ mutant (red line). C57BL/6J mice were treated for 5 consecutive days with a daily subcutaneous injection of G-CSF. At day 3 of the G-CSF treatment, recombinant KC and MIP-2 proteins (1μg/mouse, each) were i.n. administered to the G-CSF-treated mice (blue line). n = 5 for each group of mice. (TIF) [file ppat.1004893.s001.tif]

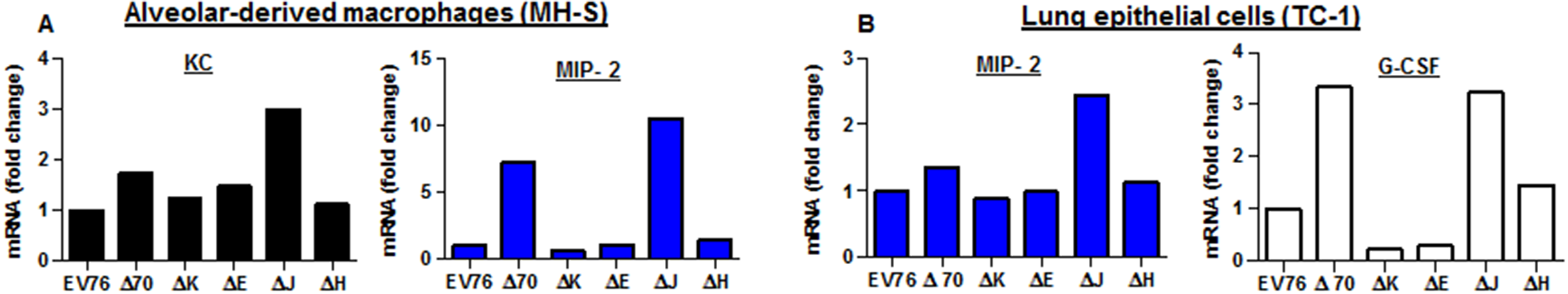

Supplement: S2 Fig — In-vitro infection of alveolar macrophages (MH-S) (A) and lung-derived epithelial cell lines (TC-1) (B) with 50 MOI of the attenuated Y. pestis strain EV76 and its Yop-depleted derivatives: EV76ΔYopK (ΔK), EV76ΔYopE (ΔE), EV76ΔYopJ (ΔJ), EV76ΔYopH (ΔH) and EV76ΔpCD1 (Δ70). The mRNA levels of the chemokines KC (A), MIP-2 (A and B) and G-CSF (B) were quantified using qPCR analysis and are presented as the fold change relative to the wild-type Y. pestis EV76. (TIF) [file ppat.1004893.s002.tif]

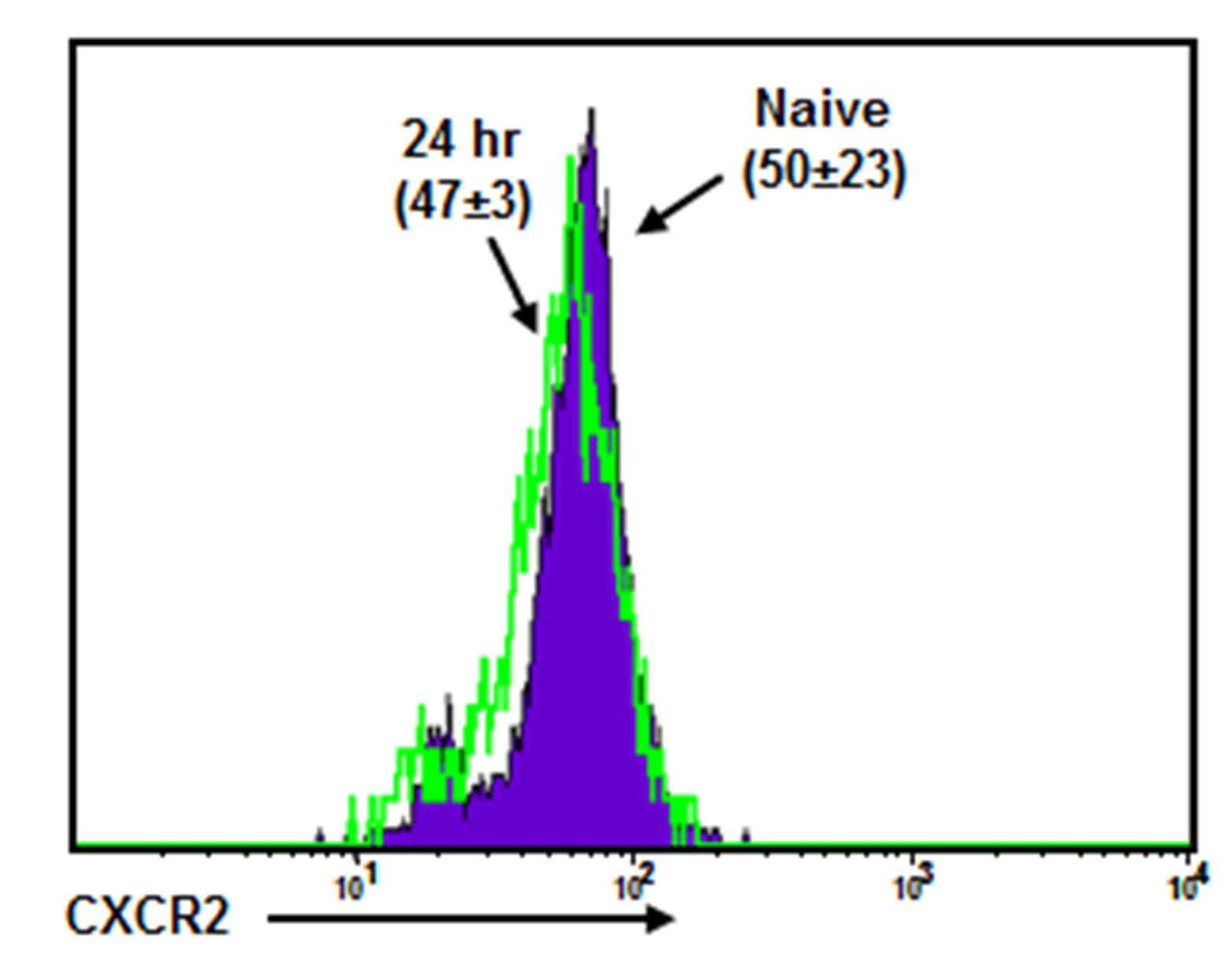

Supplement: S3 Fig — Expression of cell surface CXCR2 on circulation neutrophils isolated from the peripheral blood of C57BL/6 mice at 24 hpi with 1×10^5^ cfu of the virulent Y. pestis strain Kim53 in comparison to naive mice. Representative FACS histogram analysis showing CXCR2 expression on Gr-1high/CD11b+ peripheral blood neutrophils at 24 hpi (purple area), compared to naïve mice (green line). The average CXCR2 Geo-mean levels are indicated. (TIF) [file ppat.1004893.s003.tif]

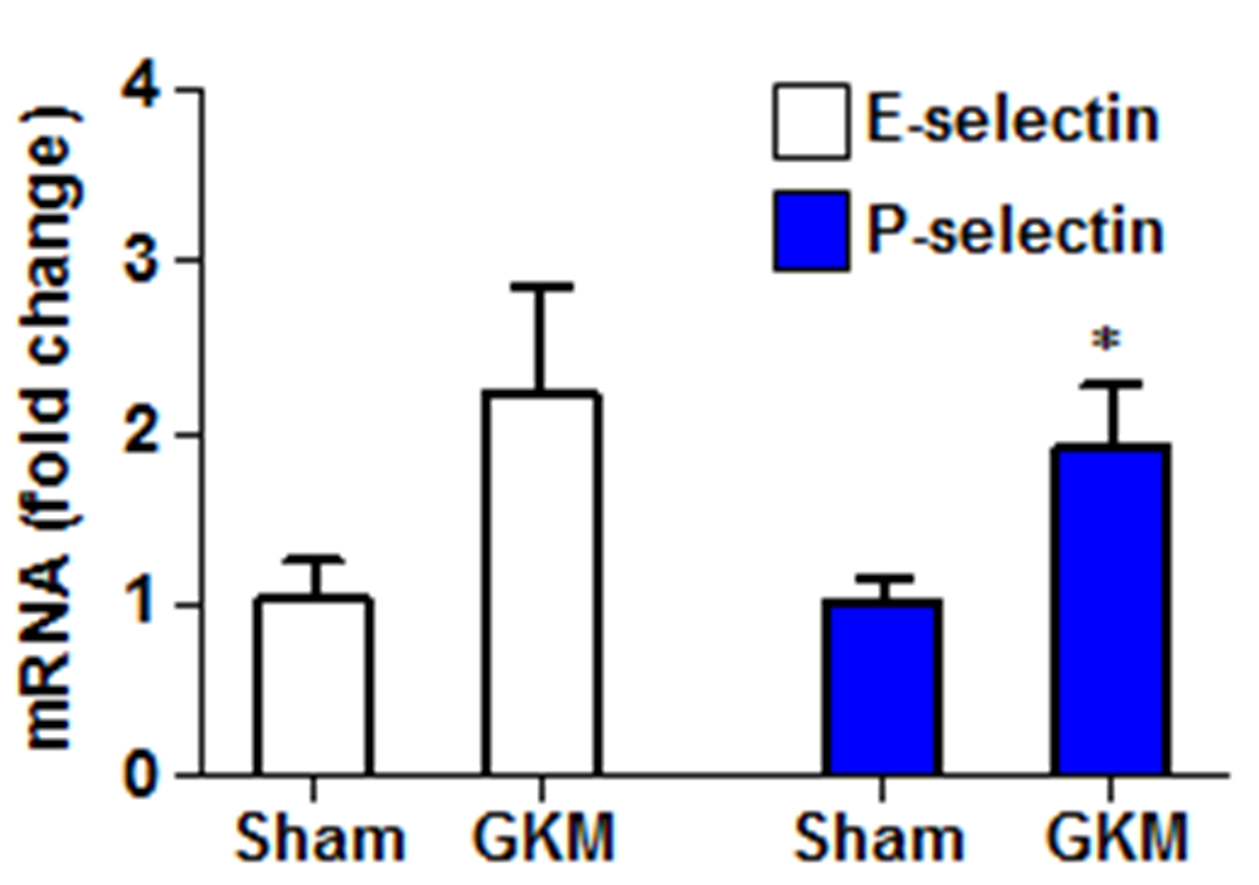

Supplement: S4 Fig — C57BL/6 mice were infected i.n. with 1×10^5^ cfu (100 LD50) of the virulent Y. pestis strain Kim53. The mRNA of sham and GKM—treated mice was purified from the infected lungs at 24 hpi and subjected to qPCR analysis of E/P-selectin gene expression. The results are presented as the means ± SEM (*p<0.05). mRNA levels are presented as fold change relative to sham-treated mice. (TIF) [file ppat.1004893.s004.tif]
